# Rice availability and stability in Africa under future socio-economic development and climatic change

**DOI:** 10.1038/s43016-023-00770-5

**Published:** 2023-06-19

**Authors:** Koen De Vos, Charlotte Janssens, Liesbet Jacobs, Benjamin Campforts, Esther Boere, Marta Kozicka, Petr Havlík, Christian Folberth, Juraj Balkovič, Miet Maertens, Gerard Govers

**Affiliations:** 1 Department of Earth and Environmental Sciences, KU Leuven, Heverlee, Belgium; 2 Research Foundation Flanders, Brussels, Belgium; 3 Biodiversity and Natural Resources Program, International Institute for Applied Systems Analysis, Laxenburg, Austria; 4 Institute for Biodiversity and Ecosystem Dynamics, University of Amsterdam, Amsterdam, the Netherlands; 5 Department of Earth Sciences, VU University Amsterdam, Amsterdam, the Netherlands

**Keywords:** Climate-change impacts, Agriculture, Projection and prediction, Socioeconomic scenarios

## Abstract

As Africa is facing multiple challenges related to food security, frameworks integrating production and availability are urgent for policymaking. Attention should be given not only to gradual socio-economic and climatic changes but also to their temporal variability. Here we present an integrated framework that allows one to assess the impacts of socio-economic development, gradual climate change and climate anomalies. We apply this framework to rice production and consumption in Africa whereby we explicitly account for the continent’s dependency on imported rice. We show that socio-economic development dictates rice availability, whereas climate change has only minor effects in the long term and is predicted not to amplify supply shocks. Still, rainfed-dominated or self-producing regions are sensitive to local climatic anomalies, while trade dominates stability in import-dependent regions. Our study suggests that facilitating agricultural development and limiting trade barriers are key in relieving future challenges to rice availability and stability.

## Abstract (web summary)

African rice production is facing high spatiotemporal variability in rice yields and uncertain supply chains. This study proposes a framework to assess the future impacts of socio-economic development and climate change on African rice availability and stability. Both local and trade-propagated climatic variabilities are important to identify future challenges.

## Main

Rice is an increasingly important staple crop in Africa. Demand quadrupled from around 10 Mt to 40 Mt between 1990 and 2018 due to rapid population growth and dietary shifts (from 7% to 9% of the caloric intake)^1,2^. Despite recent yield advances, Africa’s rice production has been lagging behind demand, making the continent increasingly import-dependent^2,3^. According to data from the Food and Agriculture Organization, up to 30% of the rice necessary to meet demand is currently imported from Southeast Asia (SEA) (17.8%) and India (12.0%). This import dependency makes Africa vulnerable to external supply and price shocks, as demonstrated during the 2008 food crisis when rice prices spiked more dramatically than those of other cereals in Africa^4^. While self-sufficiency policies could alleviate foreign effects and would allow domestic production to develop^5,6^, it is uncertain whether they would improve food security. African rice production is currently characterized by high spatiotemporal variability in rice yields^7^, and intensification is hampered by underdeveloped supply chains and the slow dissemination of higher-yielding varieties^8,9^, resulting in an uncertain supply and a price volatility of rice and food in general that exceeds that on any other continent^10^. These uncertainties, in combination with a prevalence of undernourishment of 21% in 2020, make it imperative to analyse future challenges posed by climate and socio-economic changes for the food supply in Africa. Given its growing share in Africa’s food basket, rice is of key importance in this effort^11^.

Building an integrated outlook on rice availability critically depends on plausible predictions of future rice yields. Global changes in yield alter the food system by shifting comparative advantages across trade partners, thereby changing commodity prices^12^ and rice availability. Existing studies vary in quantifications of the impact and recommended adaptation measures because of the varying scales, scenarios and assumptions used. Van Oort and Zwart^13^ found that, depending on the climate change scenario, African rice yields could range between −24% and +18% by 2070 compared with 2000, and they highlighted the importance of farmers adopting heat-resistant cultivars with a higher temperature sum. Schleussner et al.^14^ predicted limited yield losses (<5%) for sub-Saharan Africa and indicated the importance of CO_2_ fertilization to offset negative climatic effects. Gérardeux et al.^15^ projected significant rainfed rice yield losses for Senegal, which could be partially negated by adopting new cultivars, and they highlighted the importance of atmospheric CO_2_ levels ([CO_2_]). Knox et al.^16^, in contrast, found no significant impacts of climate change. Due to Africa’s high import dependency for rice, its rice availability could also be indirectly affected by climate change impacts in exporting regions, but research in this area is limited^17,18^.

Climate change affects not only gradual changes in yield but also year-to-year variability—something that is often neglected in food security outlooks^19^. Projections suggest an increase in year-to-year yield variability for wheat over 60% of its globally harvested area under climate change^20^. An increase in yield variability has also been projected for rice in Indonesia^21^ and the Philippines^22^. Despite ambitions to stabilize the African rice system, the role of climate-driven anomalies in future rice yields in Africa at present is not well understood. An important outstanding question is how the stability of the African rice system—which is defined as the temporal dimension of the availability, access and utilization of rice—will evolve under different socio-economic or climatic challenges. Moreover, it is unclear how vulnerable African regions will be to local or foreign climatic anomalies in the future for rice or any other crop.

Climatic effects will co-occur with socio-economic development. Recent efforts within the Shared Socio-economic Pathway (SSP) framework^23^ have enabled research on the response of agri-food systems to combined climatic and socio-economic futures^24^. While studies applying this framework provide insights into socio-economic or climatic effects (or a combination of both) on agricultural productivity at a global scale, they do not often consider yield variability or consumer responses, nor do they discuss regional or crop-specific aspects—all of which are integral to policymaking.

Here we present an ex ante assessment of rice availability and the stability thereof for Africa using a modelling sequence consisting of an established global economic model (Global Biosphere Management Model (GLOBIOM)^25,26^) and an established crop model (Environmental Policy Integrated Climate Model (EPIC-IIASA)^27^) for 2050. An envelope of socio-economic and climatic pathways was used to assess the integrated impacts of gradual climate change, year-to-year variations and socio-economic development on rice availability using a regional aggregation following Janssens et al.^26^. The model and scenario details can be found in the Methods. This sequential modelling approach allows us to determine both projected biophysical changes and consumer responses—including those related to (variations in) expected rice supply by Africa’s largest external supplier, SEA. We used rice consumption per capita as a proxy for rice availability and analysed the impact of both gradual changes by 2050 and year-to-year variation in yields between 2035 and 2065. By considering both trends and shocks, we gain insight into the relative importance of climate anomalies in the total variation in rice availability. Although our assessment is restricted to rice in Africa—at a level of detail relevant for effective policymaking—our framework could act as a benchmark for future food security assessments in Africa and beyond.

## Results

### Rice availability for the African rice system in the 2050s

**Fig. 1:**
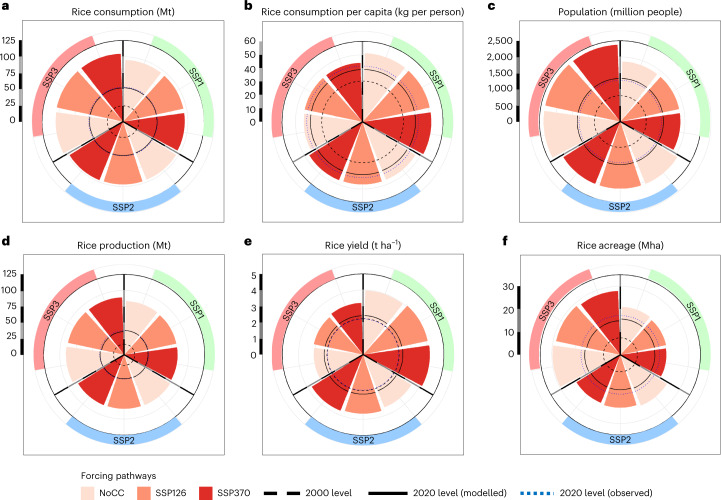
Ensemble average continental projections for the African rice system for the 2050s under different SSP × forcing scenarios. **a**, Projected total rice consumption in Mt. **b**, Projected per capita consumption in kg per person. **c**, Projected population in million people. **d**, Projected rice production in Mt. **e**, Projected rice yields in t ha^−1^. **f**, Projected rice extent in Mha. The 2000 levels represent the observed values, which also correspond to the initial state of the modelling sequence. More information on model bias and starting conditions can be found in Supplementary Table 2.

Rice consumption levels are projected to increase between the 2020s and 2050s for all scenario combinations, with considerable differences between SSP pathways (Fig. 1a). Although this increase is determined by both population growth and per capita consumption shifts (Fig. 1b–c), the former is more important. Under SSP1–NoCC, modest population growth (+40%) limits the increase in demand (+83%) despite having the highest per capita consumption (+31%) (Fig. 1b). Under SSP3–NoCC, per capita consumption growth is limited (+13%), but rapid population growth (+79%) leads to the highest rice demand increase (+101%). SSP2–NoCC, with moderate population (+56%) and per capita consumption growth (+19%), results in a moderate rice demand increase (+85%).

African rice production evolves in step with rice demand across socio-economic narratives (Fig. 1d), showing that estimates of rice supply and demand are strongly interlinked irrespective of socio-economic or agronomic conditions. Yet, the scenarios differ in how rice demand is met. Under SSP1–NoCC, the gradual yield increase is the highest (+64%), while the acreage increase is the lowest (+34%)—indicating that SSP1 (Sustainability) pathways focus on yield improvements through intensification or technological change rather than acreage expansion to increase rice production (+120%). Such yield improvements reduce production costs (Supplementary Fig. 1a), which, combined with limited population growth, also lead to higher per capita consumption levels. SSP3–NoCC results in a modest increase in yield (+27%) in combination with a large acreage expansion (+90%), which is necessary for production to increase (+141%) to meet demand. This acreage expansion increases production costs and ultimately leads to very low growth of per capita consumption. SSP2–NoCC results in ‘Middle of the Road’ increases in yield (+46%), acreage (+53%) and production (+123%).

At the continental scale, the effects of gradual climate change are, on the contrary, relatively small for all supply and demand components (Supplementary Table 1), which is consistent with previous studies^13,14,16^. This is mainly because of two factors: (1) the potentially negative effects of warming and decreasing precipitation are offset by [CO_2_] effects, and (2) the GLOBIOM model allows for shifts in management system from rainfed to irrigation to occur in addition to input intensification. Support for the occurrence of both processes is provided in Supplementary Figs. 1–5. Importantly, while the effects of climate change on rice production are limited at the continental scale, there are substantial impacts on yields in some individual regions.

### Rice stability of the African rice system in the 2050s

**Fig. 2:**
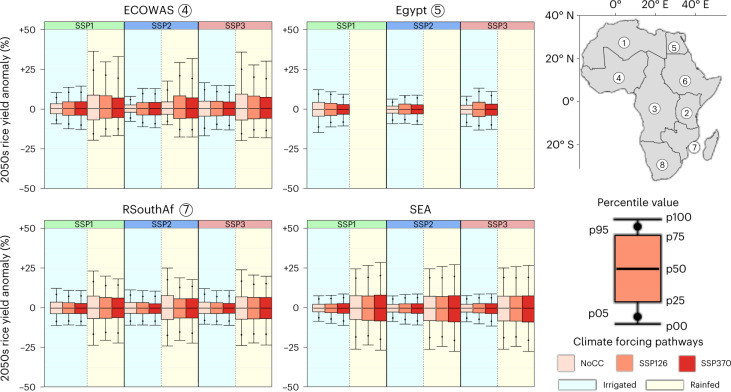
Predicted relative ensemble rice yield anomaly. Results are shown for the ECOWAS, Egypt, RSouthAf and SEA regions under different SSP × climate forcing scenarios for the 2050s compared with the expected median yield level. Values are calculated for the 2035–2065 time window (*n* = 30). For NoCC, historical variability estimates were used. A distinction is made between irrigated (blue) and rainfed (yellow) yields. Note that rainfed yields for Egypt are not included because of the limited extent of this system. Figures for the other regions can be found in Supplementary Fig. 7. The regions are numbered as follows: (1) AMU, (2) EAC, (3) ECCAS, (4) ECOWAS, (5) Egypt, (6) RCEAf, (7) RSouthAf and (8) SACU.

Anomalies in rice yield are projected to only marginally change under climate change compared to historical estimates (NoCC) (Fig. 2—for other regions, see Supplementary Fig. 7). The direction of the change, however, depends on the region, socio-economic scenario and production system and is influenced by differences in demand, rice extent and management intensity between scenarios. Yield anomalies range between −25% and 25% for rainfed production systems and are thus systematically larger than for their irrigated counterparts, for which they typically range between −15% and 15%. This difference is observed for all regions, independent of climatic or socio-economic scenario.

In SEA—Africa’s main external rice provider—anomalies in irrigated rice yield are projected to increase slightly with increasing intensity of climatic forcing (Fig. 2). This is particularly observed in the lower tails of the distribution (p00 and p05). For rainfed production systems, the signal is less clear.

**Fig. 3:**
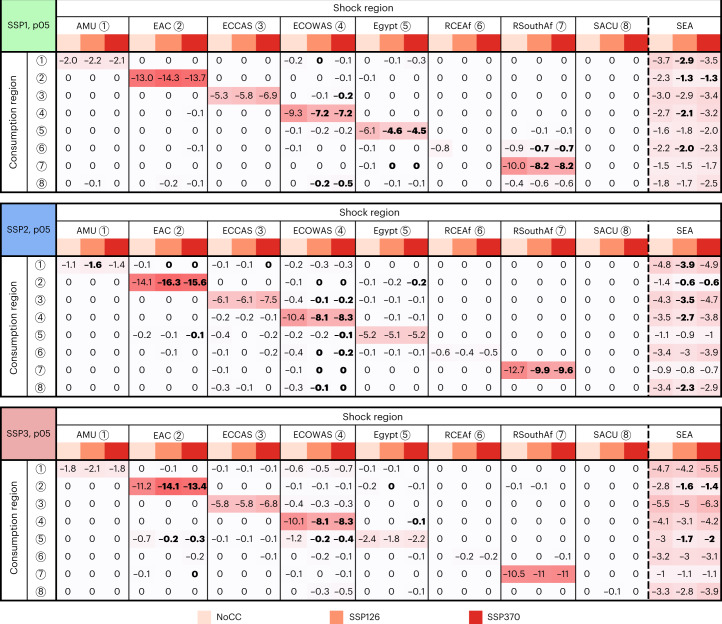
Projected consumer response. Consumer response is expressed as the relative drop in consumption (%) after a p05 yield shock occurring in the shock region (columns) under different SSP and climatic forcing pathways for the 2050s. Values in bold represent a significant difference from a NoCC forcing scenario under global climate model (GCM) spread (following a *t*-test, *n* = 5, *α* = 0.05). The effects were calculated relative to the median (Methods) and are visualized by relative shading. The regions are numbered as follows: (1) AMU, (2) EAC, (3) ECCAS, (4) ECOWAS, (5) Egypt, (6) RCEAf, (7) RSouthAf and (8) SACU. The matrices for other percentile events can be found in Supplementary Tables 3–7. Source data

The magnitude of consumer responses to climatic anomalies is not projected to change significantly under climate change when compared to historical variability (NoCC) in the majority of African regions (Fig. 3). Depending on the socio-economic scenario, we project a moderated consumer response for the Economic Community of Western African States (ECOWAS) (all SSPs), the Rest of Southern Africa (RSouthAf) (SSP1–2) and Egypt (SSP1) caused by climate change. For the Eastern African Community (EAC), we project a significant increase in the magnitude of the consumer response under SSP2–3, indicating that the EAC will become more sensitive to domestic production shocks under climate. No significant impacts of local climate change on consumption shocks could be observed for the Arab Maghreb Union (AMU), the Economic Community of Central African States (ECCAS), the Rest of Central Eastern Africa (RCEAf) or the Southern African Customs Union (SACU) (Fig. 3).

Consumption drops caused by a local yield shock (visible on the diagonal in Fig. 3) are of higher magnitude than drops due to intracontinental trade effects (visible off-diagonal), indicating that spillover effects to other African regions are limited. Local effects are pronounced in rainfed-dominated regions (such as ECOWAS and EAC) and in regions with a high self-sufficiency level (such as RSouthAf and Egypt), while they are minimal for regions with a strong reliance on imports (such as AMU and SACU). To a lesser extent, African consumers also respond to yield shocks in SEA. The magnitude of these responses is linked to the dependency on imports from SEA (Fig. 3). For some regions (such as AMU and RCEAf), the predicted consumption drops even surpass the response due to a regional shock. Under SSP370, consumption drops caused by SEA yield shocks are not significantly different from those under NoCC, while projected drops in consumption are systematically smaller under SSP126 in the majority of African regions, suggesting that reducing atmospheric forcing levels shows potential to relieve consumption drops propagated through trade.

Overall differences in consumer response between socio-economic scenarios are small. However, for all regions except Egypt and RSouthAf, the consumption response to climatic anomalies occurring in SEA is lower under SSP1 (Sustainability)—the scenario that assumes the least barriers to trade.

**Fig. 4:**
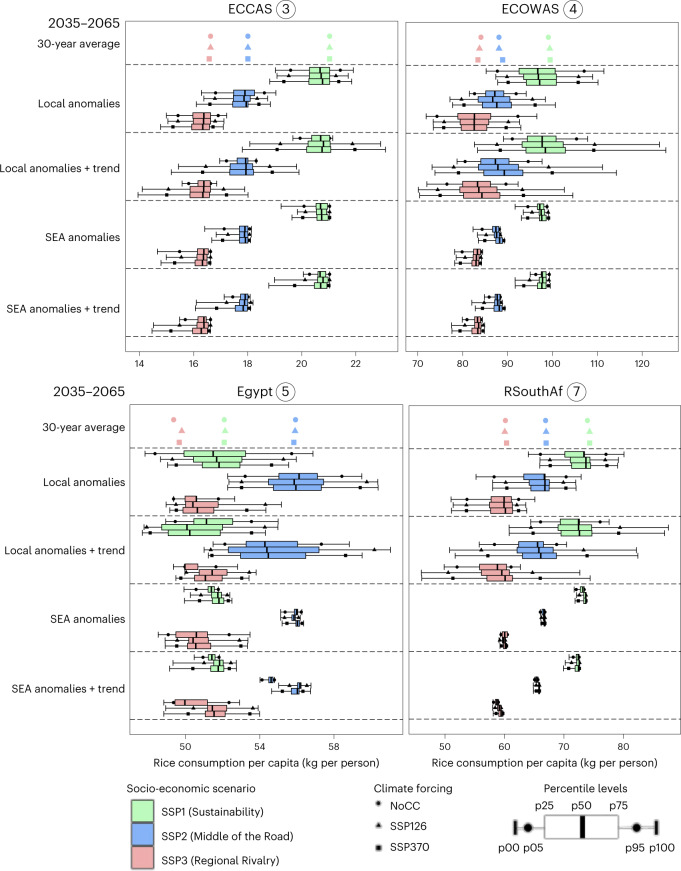
Percentile levels of projected ensemble per capita rice consumption. Results are shown in kg per person for the ECCAS, ECOWAS, Egypt and RSouthAf regions under different socio-economic and climatic narratives for the 2035–2065 time window (*n* = 30). The 30-year-average values indicate projected average per capita effects on rice consumption in the 2050s (see Fig. 1). Anomalies are calculated after linear detrending in the 2035–2065 time window. Total variation (anomalies + trend) is represented by projected values without detrending. See the Methods for the calculation of the distribution. Other regions can be found in Supplementary Fig. 8.

We observed substantial temporal variations in consumption per capita when accounting for both trends and anomalies in the 2035–2065 time window for all regions and socio-economic narratives (Fig. 4). While accounting for trends systematically increases the temporal variation compared with only considering anomalies, we also observed stark distinctions between socio-economic narratives. Local yield variations lead to effects on consumption in both the lower and upper tails of the distribution, while external (SEA) effects only translate into the lower tails. This indicates that only the negative effects of yield variation (both with and without accounting for trends) spill over to rice stability in African regions through trade (Fig. 4).

## Discussion

Consistent with other crops^28^, we found that the main driver of changes in African rice availability is the pace of socio-economic development rather than climatic pathways because of stark population effects. Through a market balance, socio-economic development also dictates rice production levels—which, together with the expected yield growth, drives pressure on land. The latter is therefore observed to be higher under SSP3 (Regional Rivalry) than under the other socio-economic pathways.

Although climate change can significantly reduce rice productivity in certain regions, this is mostly compensated for by increasing imports and/or extending production areas^29^. As the GLOBIOM model accommodates for management shifts and land allocation, we observed a more limited effect of gradual climate change than existing studies^13–15^. In particular, Van Oort and Zwart^13^ reported in some cases stronger impacts of gradual climate change but showed broadly consistent patterns, with rainfed rice production being affected more than irrigated rice production. We also predict climate change to have limited effects on temporal variations of rice yield and consumption per capita. For the majority of African regions, we observed that climate change does not increase the magnitude of rice yield shocks, contrary to what has been projected for other staple crops or for other regions^20–22^. Further research is needed to identify whether this provides opportunities for African rice production to mitigate negative climate change effects on food stability. This, however, does not imply that the African continent is safeguarded from supply shocks that can undermine rice stability—particularly as the continent’s rice availability has been observed to be vulnerable to shocks in the past^4,30^. Regions with a high degree of self-sufficiency and regions that are dominated by rainfed production systems are projected to remain equally vulnerable to local yield shocks under climate change. The vulnerability of rainfed-dominated regions is linked to their reliance on precipitation, which itself has an intrinsic variability. Although the variability in annual precipitation is expected to increase in Africa^31^, we did not observe such an increase in consumer response—rather the opposite. As yield shocks are clearly higher for rainfed production than for irrigation, the vulnerability to local yield shocks could be partially buffered through shifting to irrigation systems. However, this requires that any supplementary irrigation can be acquired sustainably (which is not achievable everywhere^32^) and demands local-level assessments.

As many African regions rely on SEA to meet their rice demand^2^, they are also affected by yield shocks occurring in the latter region. Some regions (such as AMU and SACU) are more vulnerable to climate-driven anomalies occurring in SEA than they are to regional ones. Reducing atmospheric forcing levels following SSP126 could, however, relieve the magnitude of import-driven shocks. Limiting trade barriers reduces the number of people at risk of hunger resulting from slow-onset climate change^33^, could provide a buffer in times of local shortages and—as we found—reduces the vulnerability to foreign supply shocks. Yet, after the 2008 food crisis and during the COVID-19 pandemic, the interest in self-sufficiency policies has renewed^34–36^. These crises also clearly demonstrated that supply shocks and their consequences for Africa are not limited to meteorological events or climatic anomalies alone. Also, the responses of crop yield to extreme weather events (such as severe droughts) are difficult to estimate using current crop models^37,38^. Furthermore, the GLOBIOM model does not account for cross-price effects (Methods), rendering our results a first indication rather than an accurate estimate. Future studies may further refine our estimated consumer response by accounting for spatial population dynamics such as within-country migration, in particular towards cities.

Although an array of adaptive policy measures exists that can be used to lower vulnerability to variability in the rice system^39–41^, our analysis indicates that mitigation can be done proactively by limiting socio-economic challenges. SSP1 (Sustainability) (low challenges) results in the most viable conditions in terms of per capita consumption and land use as pressures on the local production systems are limited, while SSP3 (Regional Rivalry) (high challenges) results in continued agricultural expansion and lower consumption-per-capita levels. Resilience to socio-economic or climatic challenges can be increased by agricultural productivity growth through disseminating new technologies^42,43^, improving farmers’ access to seed or credit markets^9^, or upscaling existing decision-making tools (for example, the RiceAdvice App^44^). Shifting from rainfed to irrigated production systems reduces local yield variability through limiting the effects of variability in local precipitation. This, of course, requires the availability, feasibility and accessibility of sustainable irrigation infrastructures^32,45^. Strategies that mitigate vulnerability to foreign supply shocks include upgrading domestic storage capacities and diversifying trade networks^46,47^. Although the former are already being used to buffer supply shocks (both local and external)^39,46^, it is unclear whether their capacity and level of governance are sufficient to ensure stability in the (future) African rice system. On the basis of our results, we argue for a diversification of the rice supply—whether from local production or from imports—joined by a limitation of trade barriers to relieve potential risks to the stability of rice availability.

## Conclusion

Ex ante assessments that integrate both availability and stability (of any commodity) aid in identifying obstacles within the agri-food system under global change and are therefore urgently needed to improve African food security under the future challenges the continent is facing. The approach presented here allows us to consider plausible global socio-economic and climatic futures, while still recognizing regional and crop-specific contexts. For the case of rice in Africa, we highlight the need for agricultural productivity growth as well as a careful consideration of the effects of year-to-year yield variability and the related costs and benefits of different supply strategies in future food security policymaking. Our framework integrates socio-economic development and climatic change and can serve as a basis to inform future research or policymaking, also for other commodities and regions, towards food security.

## Methods

### Modelling sequence

We used a crop (EPIC-IIASA^27^) and partial equilibrium economic (GLOBIOM) modelling sequence. The EPIC-IIASA-based modelling system is a globally gridded crop model implemented on a 5′ spatial grid. It was run herein with daily projections for solar radiation, minimum and maximum temperatures, precipitation, and relative humidity stemming from five distinct GCMs that can be considered structurally independent and representative of the range of equilibrium climate sensitivities within the Coupled Model Intercomparison Project Phase 6 (CMIP6)^48^: GFDL-ESM4^49^, IPSL-CM6A-LR^50^, MPI-ESM1-2-HR^51^, MRI-ESM2-0^52^ and UKESM1-0-LL^53^. This was done for two forcing pathways (SSP126 and SSP370) with their respective trajectories of annual atmospheric [CO_2_]. These projections are bias-corrected with the observational GSWP3W5E5 v.1.0 dataset and [CO_2_] for the forcing pathways^54–56^. The yields of four major crops (rice, wheat, maize and soybean) were thoroughly calibrated within the EPIC-IIASA model using the best available information on cropping calendar, fertilizer use, soil data and agro-ecological zoning, and they show good agreement with the spatial patterns of reported yields at the global scale^27,57^. The model’s predicted year-to-year variability of rice yields compares well to those of other global crop models^58^. Despite the fact that we are focusing on rice in Africa in this research, it is important to also consider how yields of other crops are evolving to correctly represent any spatial allocation due to shifting comparative advantages. To avoid using different models for each crop (which would require considering model bias explicitly), we opted to use EPIC-IIASA for all crop yield estimations. Detailed information on model structure and key processes is documented in Sharpley and Williams^59^, Williams^60^ and Izaurralde et al.^61^.

GLOBIOM is a spatially explicit economic partial equilibrium model of the global agriculture, forestry and bioenergy sectors. At each 10 yr time step between 2000 and 2050, the model recursively maximizes welfare (the sum of consumer and producer surplus) by adjusting production, consumption and trade patterns. Land management is governed in 212,707 Simulation Units (globally) that are delineated by altitude, slope, soil and agro-ecological classes, country borders, and a 5′ spatial resolution grid (corresponding to 10 km × 10 km at the equator). Land management is divided into four systems: irrigated, high-input rainfed, low-input rainfed and self-subsistence farming. Each management system has its own potential yields and input requirements. Although the model allows both land allocation and management shifts (for example, from high-input rainfed to irrigated) to occur within each Simulation Unit, changes in extent and shifts in management are both constrained to better reflect the inertia of changes in land cover and land use. Changes in agronomic conditions (for example, shifting sowing dates or adopting new cultivars) are not explicitly taken into account. Although crop demand was adjusted endogenously through an isoelastic demand function of product prices and exogenous projections of population and gross domestic product stemming from the socio-economic narratives for all crops, we focused only on rice in our description. Considering effects on demand for the other crops was necessary to also take into account their effects on land allocation. Direct demand-side substitution effects between crops through cross-price elasticities were not considered. Trade was modelled via spatial price equilibrium assuming homogeneous goods and nonlinear trade costs. Detailed information on the model structure, data and parameters of GLOBIOM is documented in Havlík et al.^25^ for supply and demand and Janssens et al.^26^ for trade and the specific adaptations done to better represent the African agricultural context.

### Scenario design

In this study, we used scenarios for climatic and socio-economic futures based on the SSP framework^23^. Projections on forcing scenarios come from CMIP6 for an ensemble of the GFDL-ESM4^49^, IPSL-CM6A-LR^50^, MPI-ESM1-2-HR^51^, MRI-ESM2-0^52^ and UKESM1-0-LL^53^ global climate models and were extracted from the ISIMIP3b repository as the basis for yield projections using the EPIC-IIASA crop model. In our analysis, we approached projections for different future climatic pathways merely as forcing pathways, rather than assuming that they have a socio-economic component as well. We did this deliberately to be able to disentangle socio-economic and climatic effects in the assessment and to provide an ex ante assessment outside of the scope of currently available scenarios within ISIMIP3b. NoCC represents a present climate and should be regarded as a forcing scenario without climate change; it is based on observations within the GSWP3W5E5 v.1.0 dataset^56^. SSP126 represents a forcing pathway where the global mean temperature is kept well under 2 °C warming^62^, whereas SSP370 represents a high-forcing scenario (7.0 W m^−2^ by 2100) with a particular amount of tropospheric aerosol emissions. SSP370 is a response to the gap between RCP6.0 and RCP8.5 existing prior to CMIP6 and can act as an unmitigated business-as-usual scenario^63^. Only scenarios where [CO_2_] changes over time were considered to be able to account for CO_2_ fertilization effects.

Projections on socio-economics—including population, gross domestic product, trade facilitation and dietary preferences—were taken from the SSP framework for SSP1 (Sustainability), SSP2 (Middle of the Road) and SSP3 (Regional Rivalry)^23^. These narratives represent various levels of socio-economic challenges to adaptation and mitigation to climate change. SSP1 (Sustainability) represents low challenges in both adaptation and mitigation, SSP2 (Middle of the Road) represents medium challenges, and SSP3 (Regional Rivalry) represents high challenges^23^. SSP4 (Inequality) and SSP5 (Fossil-Fuelled Development) were not considered in this study. Under SSP1 (Sustainability), economic growth is high^64^, population growth is low^65^, yield technology advances rapidly and trade barriers are limited, while there is promotion of sustainable development in terms of consumption^66^. Under SSP2 (Middle of the Road), economic and population growth are moderate^64,65^, technological advances in yield follow the Food and Agriculture Organization agricultural outlook, and current trade tariffs and subsidies are assumed^66^—hence, it can act as a business-as-usual narrative. Under SSP3 (Regional Rivalry), economic growth is low^64^, population growth is high for developing countries and low for developed countries^65^, crop yield improves slowly, and trade is restricted to aim for agricultural self-sufficiency^66^. While the climatic effects are crop-dependent, the socio-economic effects are not.

This resulted in a matrix of 45 scenario combinations (5 GCMs × 3 SSPs × 3 climate forcings), resulting in 45 different projections by the 2050s. This was reduced to 9 distinct scenario combinations (3 SSPs × 3 climate forcings) by taking the average of the projections from the different GCMs and referring to it as the ensemble projections. This approach also allowed us to perform statistical analyses explicitly using the spread between GCM projections (for example, Fig. 3 and Supplementary Tables 3–7). The potential crop yield (${Y}_{c}^{{\mathrm{p}}}$) for crop *c* at each decadal time step *T* for each simulation unit *i* under each scenario combination was calculated through multiplying both climatic (*λ*^cc^) and socio-economic (*λ*^se^) effects by the base year crop yield (*Y*_*c*,base_) (equation (1)). For the NoCC scenarios, the climatic factor was set to 1:

1 $${Y}_{c,T,i,{{\mathrm{RCP}}},{{\mathrm{GCM}}},{{\mathrm{SSP}}}}^{{\mathrm{p}}}={Y}_{c,{{\mathrm{base}}},i}\times {\lambda }_{c,T,i,{{\mathrm{RCP}}},{{\mathrm{GCM}}}}^{{{\mathrm{cc}}}}\times {\lambda }_{T,i,{{\mathrm{SSP}}}}^{{{\mathrm{se}}}}$$

Although the EPIC-IIASA crop model provides annual yield projections, the decadal resolution of the GLOBIOM economic model constrains the modelling sequence to the latter resolution, making it suitable for only long-term projections. To convert the annual EPIC-IIASA output to the decadal GLOBIOM resolution, we used 30 yr average values—that is, 15 yr before and 15 yr after—for the years 2000–2050 (equation (2)). The historical (base) yields (*Y*_*c*,base_) were calibrated using the Food and Agriculture Organization yields for 2000, while the gradual climatic effects of the year 2050 (${\lambda }_{2050}^{{\mathrm{c}}}$) (Supplementary Figs. 2 and 3) were calculated by taking the average effect between 2035 and 2065:

2 $${\lambda }_{c,T,i,{{\mathrm{RCP}}},{{\mathrm{GCM}}}}^{{{\mathrm{cc}}}}=\frac{1}{{Y}_{c,{{\mathrm{base}}},i,{{\mathrm{RCP}}},{{\mathrm{GCM}}}}}\mathop{\sum }\limits_{t=T-15}^{T+15}\frac{{Y}_{c,t,i,{{\mathrm{RCP}}},{{\mathrm{GCM}}}}}{30}=\frac{{\bar{Y}}_{c,T,i,{{\mathrm{RCP}}},{{\mathrm{GCM}}}}}{{Y}_{c,{{\mathrm{base}}},i,{{\mathrm{RCP}}},{{\mathrm{GCM}}}}}$$

### Temporal variation

In our analysis, we used two different measures to identify variation in rice availability within the 2035–2065 time window: (1) when only considering yield anomalies and (2) when also accounting for trends existing within the time window. For both approaches, we used a percentile framework. To model temporal variations smaller than the fixed decadal resolution of the GLOBIOM model, we first ran the modelling sequence from 2000 to 2050 (as also established by previous research^12,33,67^) to provide long-term, gradual values. After this standard run, an additional time step was performed without altering the socio-economic conditions (for example, population and gross domestic product) and by constraining both expansion/abandonment and management shifts—mimicking the fact that a producer would be unaware of existing temporal variations.

To calculate the total variation between 2035 and 2065 (trend + anomaly), a supplementary factor (*λ*^var^) that reflects the annual variation in rice yield as modelled by the EPIC-IIASA crop model (equation (3)) was added to equation (1) during the additional time step. The value of this supplementary factor is based on a percentile value (p*X*) relative to the average within the same 30 yr time window (equation (4)). In this study, we used the p00, p05, p25, p50, p75, p95 and p100 percentile levels (p*X*) and restricted ourselves to the 1985–2015 and 2035–2065 time windows to represent historical variations and variation for the 2050s, respectively:

3 $$\begin{array}{l}{Y}_{{{\mathrm{rice}}},\,T,i,{{\mathrm{RCP}}},{{\mathrm{GCM}}},{{\mathrm{SSP}}},{{\mathrm{p}}X}}^{{\mathrm{p}}}={Y}_{{{\mathrm{rice}}},{{\mathrm{base}}},i}\times {\lambda }_{{{\mathrm{rice}}},T,i,{{\mathrm{RCP}}},{{\mathrm{GCM}}}}^{{{\mathrm{cc}}}}\\\times {\lambda }_{T,i,{{\mathrm{SSP}}}}^{{{\mathrm{se}}}}\times {\lambda }_{{{\mathrm{rice}}},T,i,{{\mathrm{RCP}}},{{\mathrm{GCM}}},{{\mathrm{p}}X}}^{{{\mathrm{var}}}}\end{array}$$

4 $${\lambda }_{{{\mathrm{rice}}},T,i,{{\mathrm{RCP}}},{{\mathrm{GCM}}},{{\mathrm{p}}X}}^{\mathrm{var}}=\frac{{Y}_{{{\mathrm{rice}}},T,i,{{\mathrm{RCP}}},{{\mathrm{GCM}}},{{\mathrm{p}}X}}}{\frac{1}{30}\mathop{\sum }\limits_{T-15}^{T+15}{Y}_{{{\mathrm{rice}}},t,i,{{\mathrm{RCP}}},{{\mathrm{GCM}}}}}=\,\frac{{Y}_{{{\mathrm{rice}}},T,i,{{\mathrm{RCP}}},{{\mathrm{GCM}}},{{\mathrm{p}}X}}}{{\bar{Y}}_{{{\mathrm{rice}}},T,i,{{\mathrm{RCP}}},{{\mathrm{GCM}}}}}$$

To estimate the expected yield anomaly, we used a similar approach (equation (5)). Our supplementary factor (*λ*^anom^) is now—depending on the significance of the trend existing in the time windows (*p*^*α*^)—either (1) determined by the residual terms (*ε*^*α*^) of a linear detrended time series of annual yield existing in the time window (equation (6)) or (2) considered equal to the total variation (equation (7)):

5 $$\begin{array}{l}{Y}_{{{\mathrm{rice}}},\,T,i,{{\mathrm{RCP}}},{{\mathrm{GCM}}},{{\mathrm{SSP}}},{{\mathrm{p}}X}}^{{\mathrm{p}}}={Y}_{{{\mathrm{rice}}},{{\mathrm{base}}},i}\times {\lambda }_{{{\mathrm{rice}}},T,i,{{\mathrm{RCP}}},{{\mathrm{GCM}}}}^{{{\mathrm{cc}}}}\\\times \,{\lambda }_{T,i,{{\mathrm{SSP}}}}^{{{\mathrm{se}}}}\times {\lambda }_{{{\mathrm{rice}}},T,i,{{\mathrm{RCP}}},{{\mathrm{GCM}}},{{\mathrm{p}}X}}^{{{\mathrm{anom}}}}\,\end{array}$$

6 $${Y}_{{{\mathrm{rice}}},t,i,{{\mathrm{RCP}}},{{\mathrm{GCM}}}}^{{\prime} }={\alpha }_{0,{{\mathrm{rice}}},i,{{\mathrm{RCP}}},{{\mathrm{GCM}}}}+{\alpha }_{1,{{\mathrm{rice}}},i,{{\mathrm{RCP}}},{{\mathrm{GCM}}}}\,t+{\varepsilon }_{{{\mathrm{rice}}},t,i,{{\mathrm{RCP}}},{{\mathrm{GCM}}}}^{\alpha }$$

7 $${\lambda }_{{{\mathrm{rice}}},T,i,{{\mathrm{RCP}}},{{\mathrm{GCM}}},{{\mathrm{p}}X}}^{{{\mathrm{anom}}}}=\,\left\{\begin{array}{c}{p}_{{{\mathrm{rice}}},i,{{\mathrm{RCP}}},{{\mathrm{GCM}}}}^{\alpha } > 0.05\to \,{\lambda }_{{{\mathrm{rice}}},T,i,{{\mathrm{RCP}}},{{\mathrm{GCM}}},{{\mathrm{p}}X}}^{\mathrm{var}}\,\\ {p}_{{{\mathrm{rice}}},i,{{\mathrm{RCP}}},{{\mathrm{GCM}}}}^{\alpha }\le 0.05\to \,\frac{1-{\varepsilon }_{{{\mathrm{rice}}},T,i,{{\mathrm{RCP}}},{{\mathrm{GCM}}},{{\mathrm{p}}X}}^{\alpha }}{{\bar{Y}}_{{{\mathrm{rice}}},T,i,{{\mathrm{RCP}}},{{\mathrm{GCM}}}}}\end{array}\right.$$

These supplementary factors are added to the equation for rice within only a single demand region at a time to identify effects of single events occurring rather than the occurrence of compound events. Variations are introduced in all African regions and in SEA, Africa’s main rice provider. This means that for all other crops and regions, supplementary factors are considered to have a value of 1—thus eliminating any effects of variation.

### Consumer responses

To identify the consumer response to these temporal variations in each demand region *i*, we used the relative difference between rice consumption per capita ($\widetilde{C}$) at p*X* and that at p50 (median) yield percentile values (equation (8)). This was done for both the total variation (for example, Fig. 4) and the expected shocks (for example, Fig. 3). Note that the tilde (~) indicates that these values are output generated by the GLOBIOM model:

8 $$\Delta {\widetilde{C}}_{{{\mathrm{rice}}},T,i,{{\mathrm{RCP}}},{{\mathrm{GCM}}},{{\mathrm{SSP}}},{{\mathrm{p}}X}}^{ \% }=\frac{{\widetilde{C}}_{{{\mathrm{rice}}},T,i,{{\mathrm{RCP}}},{{\mathrm{GCM}}},{{\mathrm{SSP}}},{{\mathrm{p}}X}}-{\widetilde{C}}_{{{\mathrm{rice}}},T,i,{{\mathrm{RCP}}},{{\mathrm{GCM}}},{{\mathrm{SSP}}},{\mathrm{p}}50}}{{\widetilde{C}}_{{{\mathrm{rice}}},T,i,{{\mathrm{RCP}}},{{\mathrm{GCM}}},{{\mathrm{SSP}}},{\mathrm{p}}50}}$$

### Reporting summary

Further information on research design is available in the Nature Portfolio Reporting Summary linked to this article.

## Supplementary information

Supplementary Information Supplementary Figs. 1–9 and Tables 1–7.

Reporting Summary

## Data Availability

The datasets generated and/or analysed during the current study are available from the corresponding author on request. Source data are provided with this paper.

## Source data

Source Data Fig. 3 Tabular version of Fig. 3.

## Code availability

The code used for the scenario analyses is available from the corresponding author on request.
