# Supplementary material for: Rice availability and stability in Africa under future socio-economic development and climatic change
Source: Nat Food. 2023 Jun 19;4(6):518–27. doi: 10.1038/s43016-023-00770-5 (PMC10289898; doi:10.1038/s43016-023-00770-5)
Supplement: Supplementary file 1 — Supplementary Figs. 1–9 and Tables 1–7. [file 43016_2023_770_MOESM1_ESM.pdf]

nature food

Article

https://doi.org/10.1038/s43016-023-00770-5

Rice availability and stability in Africa under future socio-economic development and climatic change

In the format provided by the
authors and unedited

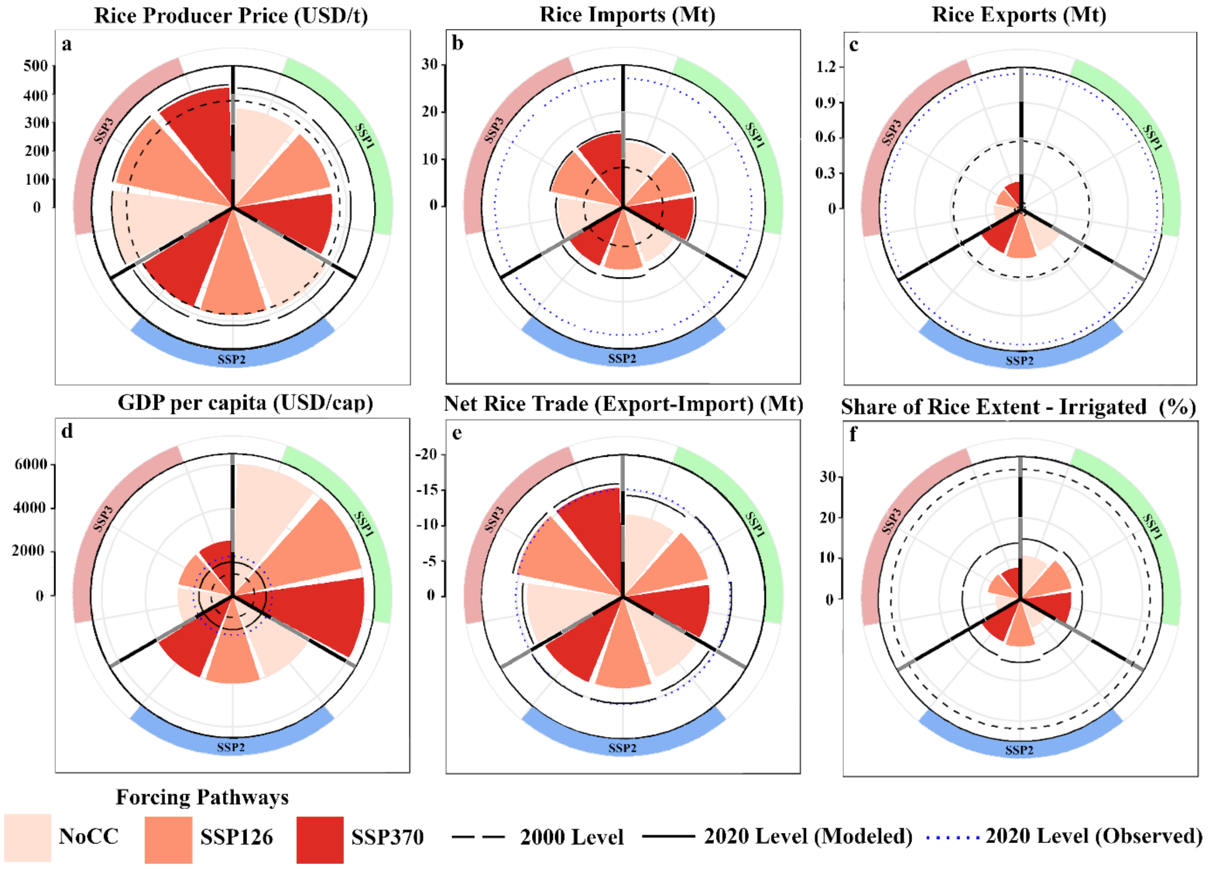

Supplementary Figure 1: ***Ensemble average continental projections for the African rice system for 2050s*** *under different SSPxForcing scenarios. a) projected rice producer price in USD per ton, b) rice imports in Mt, c) rice exports in Mt, d) gross domestic product (GDP) per capita in USD per capita, e) Net rice trade (exports-imports) in Mt. f) share of the rice extent being irrigated in %. 2000 levels represent observed values which also correspond to the initial state of the modelling sequence – more information on model bias and starting conditions can be found in Supplementary Table 2. Observed 2020 levels are presented if available.*

*Supplementary Table 1: **Ensemble long-term projections** for the different components of the African rice system for 2050s under different SSPxForcing scenarios. Numbers in brackets indicate the standard deviation between GCM models (n=5).*

2050s	*SSP1 - Sustainability*			*SSP2 – Middle of the Road*			*SSP3 – Regional Rivalry*		
	NoCC	*SSP126*	*SSP370*	*NoCC*	*SSP126*	*SSP370*	*NoCC*	*SSP126*	*SSP370*
Rice Production (Mt)	*83.56*	*82.87 (0.76)*	*82.96 (0.59)*	*84.42*	*84.05 (0.84)*	*84.43 (0.95)*	*90.54*	*89.26 (1.11)*	*89.29 (0.63)*
Rice Yield (t/ha)	*3.98*	*4.07 (0.26)*	*4.13 (0.15)*	*3.52*	*3.66 (0.19)*	*3.75 (0.17)*	*3.05*	*3.12 (0.15)*	*3.21 (0.16)*
Rice Area (Mha)	*21.01*	*20.43 (1.02)*	*20.15 (0.89)*	*23.96*	*23.09 (1.03)*	*22.60 (1.08)*	*29.66*	*28.71 (1.14)*	*27.93 (1.38)*
Rice Consumption (Mt)	*95.20*	*95.09 (0.01)*	*95.16 (0.01)*	*96.61*	*96.92 (0.14)*	*97.42 (0.42)*	*104.08*	*104.62 (0.29)*	*104.66 (0.28)*
Rice Consumption per capita (kg/cap.)	*51.18*	*51.12 (0.01)*	*51.16 (0.01)*	*46.45*	*46.60 (0.07)*	*46.84 (0.22)*	*43.92*	*44.14 (0.12)*	*44.16 (0.12)*
Rice Producer Price (USD/t)	*349.76*	*350.09 (1.09)*	*350.25 (0.80)*	*387.76*	*379.99 (2.79)*	*378.14 (2.39)*	*429.60*	*423.19 (2.21)*	*423.34 (1.92)*
Rice Import (Mt)	*25.27*	*27.36 (1.14)*	*27.42 (1.18)*	*23.25*	*24.57 (1.56)*	*24.76 (1.25)*	*25.49*	*28.82 (1.53)*	*28.86 (0.93)*
Rice Export (Mt)	*3.74*	*4.76 (0.37)*	*4.86 (0.42)*	*0.71*	*0.77 (0.02)*	*0.74 (0.04)*	*0.43*	*0.41 (0.01)*	*0.43 (0.05)*
Rice Net Trade (Mt)	*-21.54*	*-22.60 (1.35)*	*-22.57 (1.21)*	*-22.54*	*-23.81 (1.56)*	*-24.02 (1.27)*	*-25.06*	*-28.41 (1.53)*	*-28.43 (0.91)*
Rice Irrigation Share (%)	*10.76*	*12.79 (2.80)*	*12.53 (2.68)*	*7.54*	*11.68 (2.85)*	*11.24 (2.67)*	*6.17*	*8.26 (1.77)*	*7.82 (1.80)*
Population (Bln. Pers.)	*1.86*	*1.86*	*1.86*	*2.08*	*2.08*	*2.08*	*2.37*	*2.37*	*2.37*
GDP per capita (USD/cap)	*6022.24*	*6022.24*	*6022.24*	*4020.99*	*4020.99*	*4020.99*	*2541.50*	*2541.50*	*2541.50*

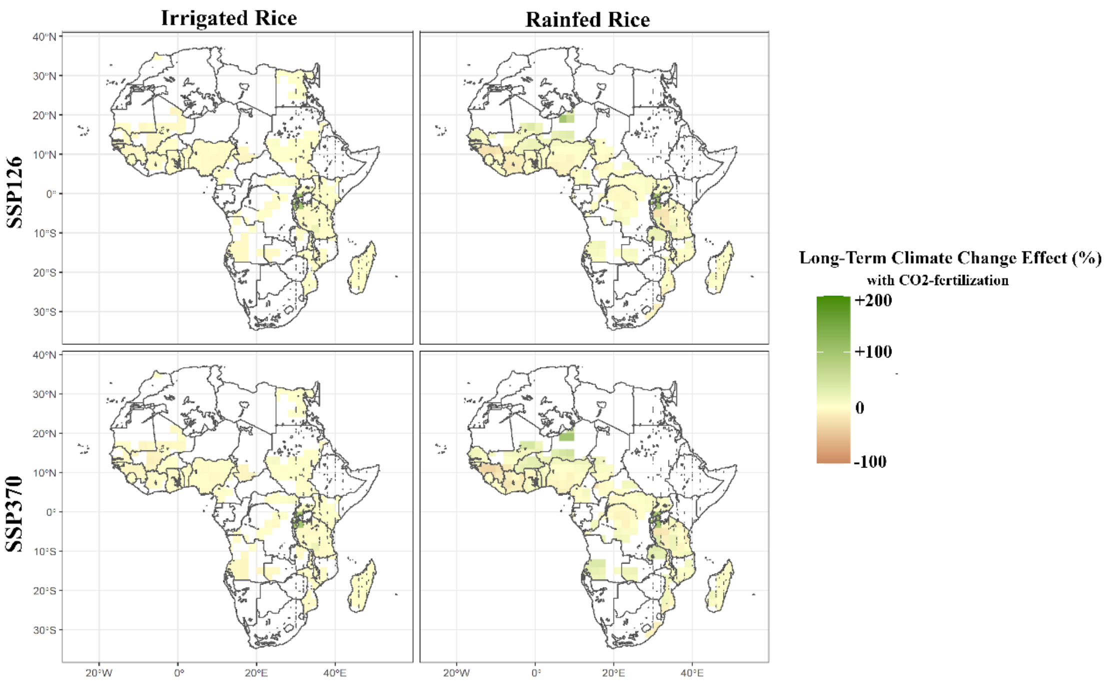

Supplementary Figure 2: ***Climate change effects on irrigated and rainfed rice yields for 2050s*** *(relative to NoCC) for both the SSP126 and SSP370 forcing scenarios as modeled by the EPIC-IIASA crop model while taking CO2-fertilization effects into account. Only production units with an area of at least 100ha in 2000 are shown. Climate effects are calculated for the 2035-2065 time window.*

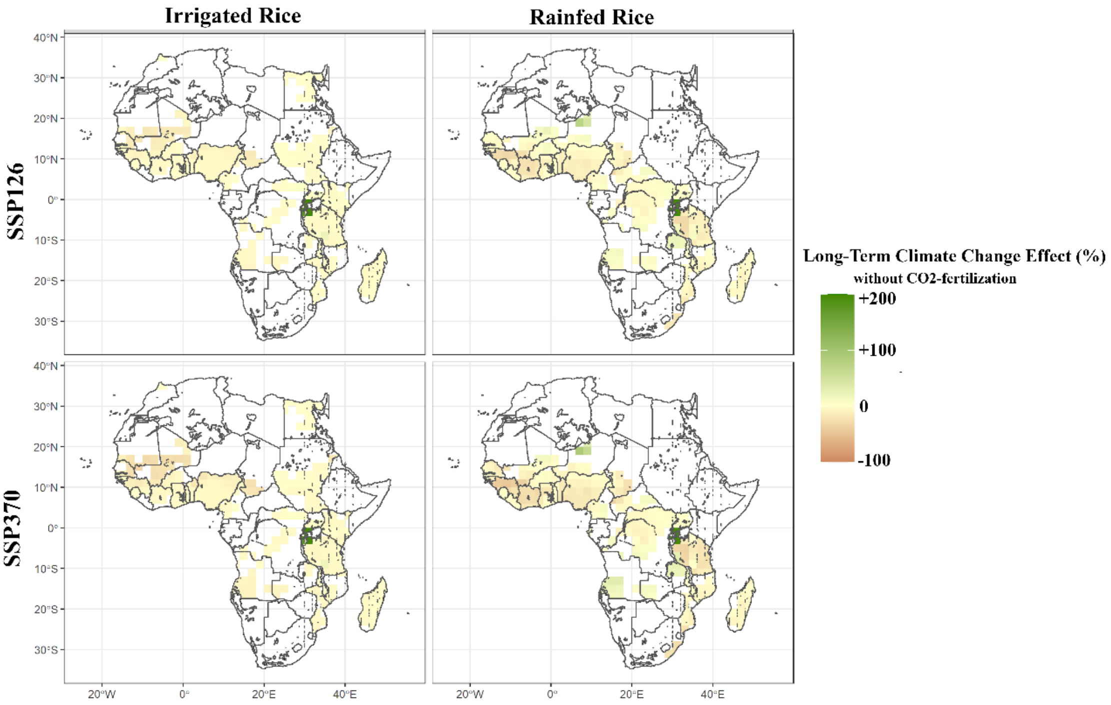

*Supplementary Figure 3: **Climate change effects on irrigated and rainfed rice yields for 2050s** (relative to NoCC) for both the SSP126 and SSP370 forcing scenarios as modeled by the EPIC-IIASA crop model without taking CO2-fertilization effects into account. Only production units with an area of at least 100ha in 2000 are shown. Climate effects are calculated for the 2035-2065 time window.*

*Supplementary Table 2: **Starting conditions and modeled vs. observed levels** for the key parameters as described through the lines in Figure 1. Observed 2020 values are taken from FAOSTAT.*

	Starting Condition (2000 Level)	Observed 2020 Level	Modeled 2020 Level (SSP2)	Model Bias
**Consumption**	24.33 Mt	53.03 Mt	52.00 Mt	-1.94 %
**Consumption per Capita**	30.44 kg/cap	41.6 kg/cap	39.03 kg/cap	-6.17 %
**Population**	799.52 Mln pers.	1274.96 Mln pers.	1332.33 Mln pers	+4.50 %
**Production**	16.55 Mt	37.89 Mt	37.72 Mt	-0.44 %
**Yield**	2.23 t/ha	2.21 t/ha	2.41 t/ha	+9.46 %
**Area**	7.42 Mha	17.17 Mha	15.62 Mha	-9.05 %

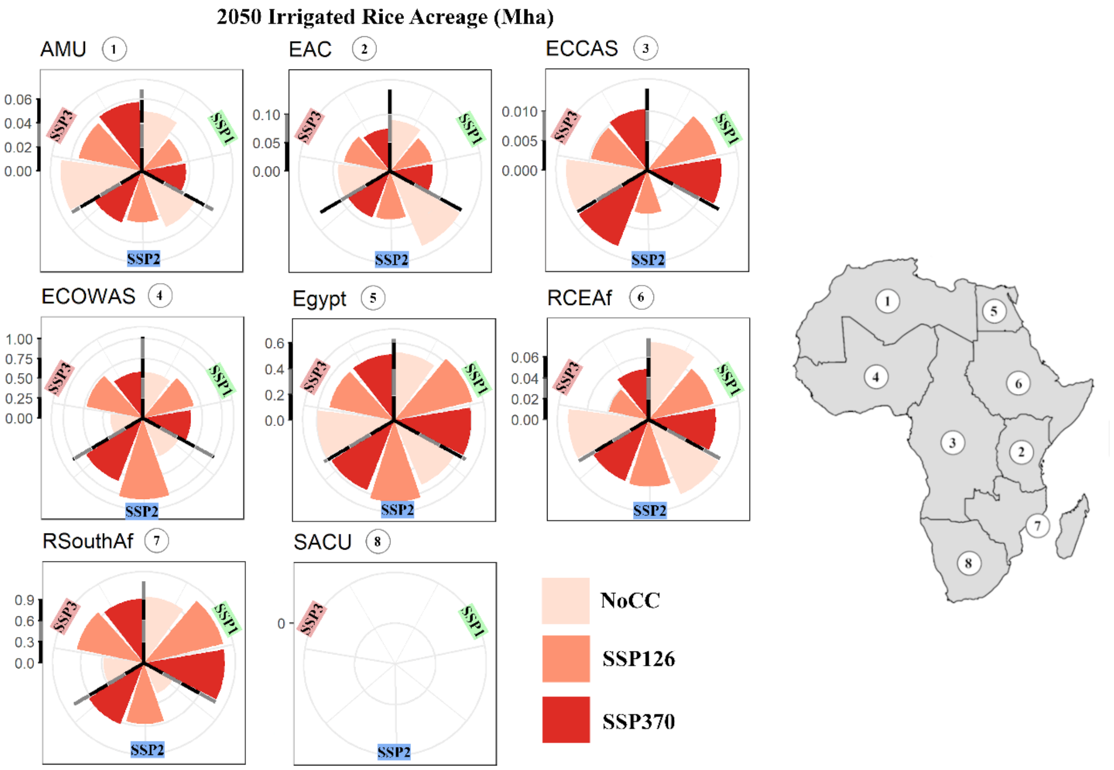

Supplementary Figure 4: ***Regional ensemble projections of irrigated rice acreage*** *(in Mha) under different SSPxForcing scenarios.* ① AMU: Arab Maghreb Union, ② EAC: Eastern African Community, ③ ECCAS: Economic Community of Central African States, ④ ECOWAS: Economic Community of Western African States, ⑤ Egypt, ⑥ RCEAf: Rest of Central Eastern Africa, ⑦ RSouthAf: Rest of Southern Africa, ⑧ SACU: Southern African Customs Union.

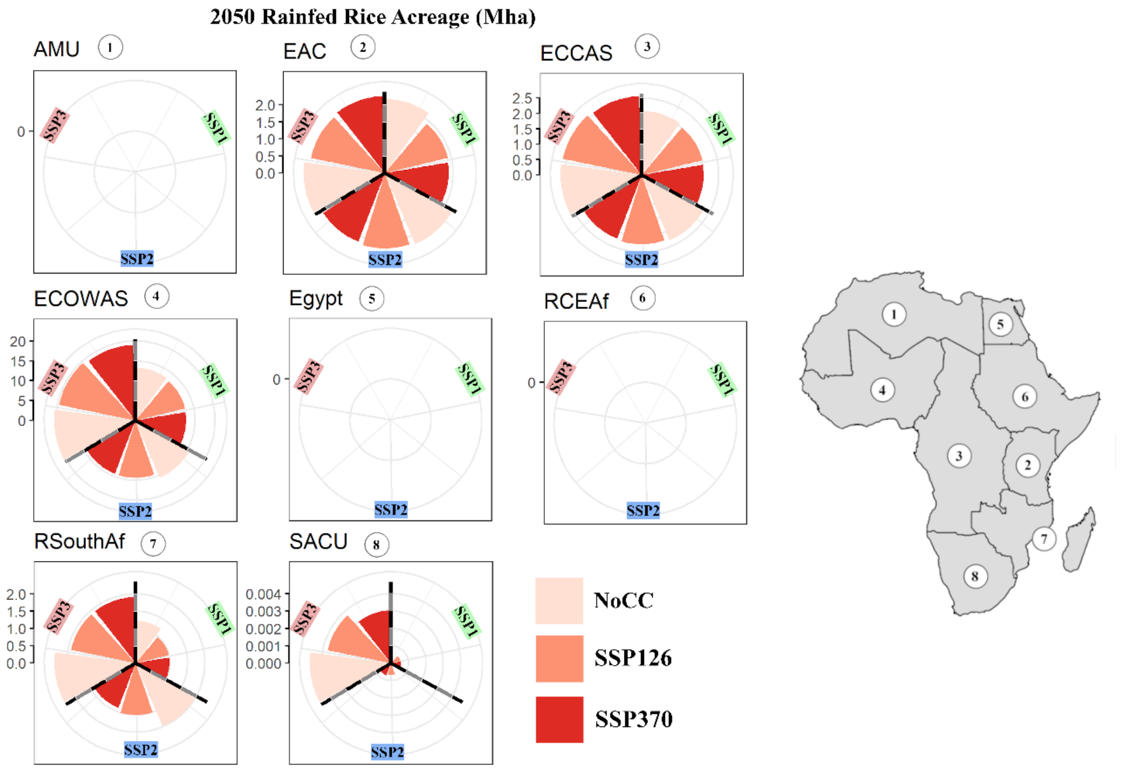

*Supplementary Figure 5: **Regional ensemble projections of rainfed rice acreage** (in Mha) under different SSPxForcing scenarios.* ① AMU: Arab Maghreb Union, ② EAC: Eastern African Community, ③ ECCAS: Economic Community of Central African States, ④ ECOWAS: Economic Community of Western African States, ⑤ Egypt, ⑥ RCEAf: Rest of Central Eastern Africa, ⑦ RSouthAf: Rest of Southern Africa, ⑧ SACU: Southern African Customs Union.

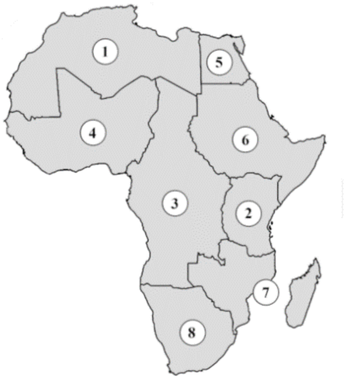

	Region	% African Supply	% Irrigation Extent	Self-Sufficiency
①	AMU	0.6	> 99.9	0.52
②	EAC	5.8	5.4	0.79
③	ECCAS	3.2	1.3	0.62
④	ECOWAS	41.6	6.0	0.69
⑤	Egypt	11.0	> 99.9	1.00
⑥	RCEAf	0.1	> 99.9	0.10
⑦	RSouthAf	10.3	54.4	0.93
⑧	SACU	< 0.1	< 0.1	< 0.00
	SEA	13.8	26.6	1.05

Supplementary Figure 6: **Regional importance of production to the continental supply, percentage of plots under irrigation and self-sufficiency** (calculated as production/consumption) for the different aggregated regions used in this study – values are given for SSP2-NoCC in the year 2020. ① AMU: Arab Maghreb Union, ② EAC: Eastern African Community, ③ ECCAS: Economic Community of Central African States, ④ ECOWAS: Economic Community of Western African States, ⑤ Egypt, ⑥ RCEAf: Rest of Central Eastern Africa, ⑦ RSouthAf: Rest of Southern Africa, ⑧ SACU: Southern African Customs Union.

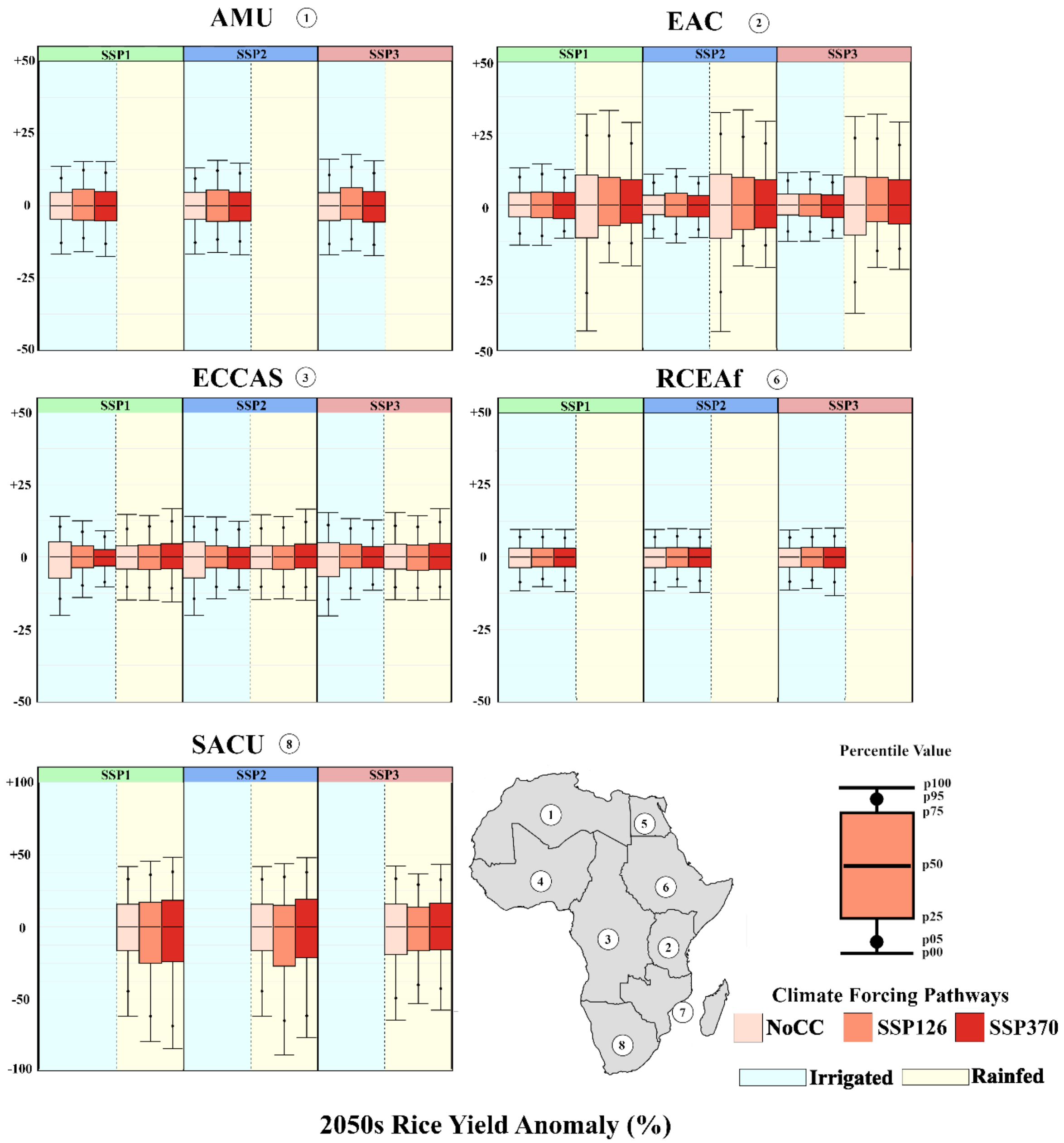

*Supplementary Figure 7: **Predicted ensemble rice yield percentile**s for the AMU, EAC, ECCAS, RCEAf, and SACU regions under different SSPxForcing scenarios for 2050s. Percentile Values are calculated for the 2035-2065 time window (n=30). Yields are separated between Irrigated (blue) and Rainfed (yellow) yields. Note that rainfed or irrigated yields for some regions are not included because of their limited extent and that the scale represented on the y-axis differs for SACU. ① AMU: Arab Maghreb Union, ② EAC: Eastern African Community, ③ ECCAS: Economic Community of Central African States, ④ ECOWAS: Economic Community of Western African States, ⑤ Egypt, ⑥ RCEAf: Rest of Central Eastern Africa, ⑦ RSouthAf: Rest of Southern Africa, ⑧ SACU: Southern African Customs Union.*

*Supplementary Table 3: **Expected relative consumption shock** in % in the affected region (rows) during a p00 yield shock in the shock region (columns) under different SSP and forcing pathways for 2050s. Values in bold represent a significant difference to a NoCC forcing scenario under GCM spread (following a t-test, n=5, alpha = 0.05). Effects are calculated relative to the median (see Methods). ① AMU: Arab Maghreb Union, ② EAC: Eastern African Community, ③ ECCAS: Economic Community of Central African States, ④ ECOWAS: Economic Community of Western African States, ⑤ Egypt, ⑥ RCEAf: Rest of Central Eastern Africa, ⑦ RSouthAf: Rest of Southern Africa, ⑧ SACU: Southern African Customs Union, SEA: Southeast-Asia.*

**SSP1 - p00**		**Shock Region**																										
		AMU ①			EAC ②			ECCAS ③			ECOWAS ④			Egypt ⑤			RCEAf ⑥			RSouthAf ⑦			SACU ⑧			SEA		
**Consumption Region**	①	-2.7	-2.4	-2.8	0	0	-0.1	0	0	0	-0.2	-0.1	-0.1	0	-0.1	-0.3	0	0	0	0	0	0	0	0	0	-6.4	**-4.2**	-5.2
	②	0	0	0	-15.9	-17.6	-16.0	0	0	0	0	-0.1	**-0.1**	-0.1	-0.1	**0**	0	0	0	0	0	0	0	0	0	-2.6	**-1.4**	**-1.4**
	③	0	0	0	0	-0.1	-0.1	-8.0	-7.9	-9.4	0	-0.1	**-0.2**	0	0	-0.1	0	0	0	0	0	-0.1	0	0	0	-7.1	**-4.3**	-5.3
	④	0	0	0	0	0	**-0.1**	0	0	0	-11.9	**-9.8**	**-9.7**	0	0	0	0	0	0	0	0	0	0	0	0	-5.7	-4.1	-4.7
	⑤	0	0	0	0	-0.1	-0.1	0	0	0	-0.2	-0.3	-0.2	-7.1	-5.9	**-5.4**	0	0	0	-0.2	-0.1	-0.2	0	0	0	-2.9	-2.9	-3.6
	⑥	0	0	0	0	0	-0.1	0	0	0	0	0	0	-0.8	**-0.1**	0	-0.8	-0.5	**-0.6**	-1.9	**-1.1**	**-1.1**	0	0	0	-4.2	-3.2	-3.6
	⑦	0	0	0	0	-0.1	0.0	0	0	0	-0.1	0	**0**	-0.2	**0**	**0**	0	0	0	-12.3	**-10.5**	**-10.8**	0	0	0	-2.0	-2.1	-2.5
	⑧	0	-0.1	0	0	**-0.3**	**-0.2**	0	0	0	0	-0.3	**-0.5**	0	-0.3	**-0.2**	0	0	0	-1.8	**-1.1**	**-1.1**	0	0	0	-3.9	**-2.5**	-3.3

**SSP2 - p00**		**Shock Region**																										
		AMU ①			EAC ②			ECCAS ③			ECOWAS ④			Egypt ⑤			RCEAf ⑥			RSouthAf ⑦			SACU ⑧			SEA		
**Consumption Region**	①	-2.0	-2.1	-1.8	-0.1	**0**	0	-0.1	-0.2	0	-0.3	-0.3	-0.3	0	0	0	0	0	0	0	0	0	0	0	0	-7.5	**-5.5**	-6.4
	②	0	0	0	-16.6	**-18.6**	-17.5	0	0	0	-0.1	**0**	**0**	-0.2	-0.2	-0.2	0	0	0	0	0	0	0	0	0	-1.5	**-0.8**	**-0.7**
	③	0	0	0	-0.4	-0.3	**-0.1**	-9.0	-8.4	-10.3	-0.5	**-0.2**	**-0.2**	-0.1	-0.2	-0.2	0	0	0	0	0	0	0	0	0	-8.3	**-5.9**	-7.0
	④	0	0	0	-0.4	-0.4	**-0.2**	0	0	0	-13.1	**-11.0**	**-11.2**	-0.1	-0.1	-0.1	0	0	0	0	0	0	0	0	0	-5.8	**-4.9**	**-5.4**
	⑤	0	0	0	-0.3	**-0.1**	**-0.1**	-0.1	0	**0**	-0.3	-0.3	**-0.2**	-6.9	-6.3	-6.6	0	0	0	0	0	0	0	0	0	-1.5	**-1.3**	-1.5
	⑥	0	0	0	0	-0.1	0	0	0	0	-0.6	**0**	**-0.2**	-0.2	**-0.1**	-0.1	-1.0	**-0.5**	**-0.6**	0	0	0	0	0	0	-6.6	**-4.3**	**-5.3**
	⑦	0	0	0	-0.3	-0.2	-0.1	0	0	0	-0.1	**0**	**0**	0	0	0	0	0	0	-17.3	**-13.2**	**-13.2**	0	0	0	-0.9	-0.9	-1.0
	⑧	0	0	0	0	0	-0.1	0	0	0	-0.3	**-0.1**	**-0.1**	0	0	0	0	0	0	0	0	0	0	0	0	-4.5	**-3.4**	**-3.7**

**SSP3 - p00**		**Shock Region**																										
		AMU ①			EAC ②			ECCAS ③			ECOWAS ④			Egypt ⑤			RCEAf ⑥			RSouthAf ⑦			SACU ⑧			SEA		
**Consumption Region**	①	-3.1	-3.0	-2.7	0	-0.1	0	-0.1	-0.1	-0.1	-0.9	-0.9	-0.9	-0.1	-0.1	0	0	0	0	0	-0.1	0	0	0	0	-7.6	**-6.1**	-7.9
	②	0	0	0	-13.3	**-16.6**	-15.9	0	0	0	-0.1	-0.1	-0.1	-0.5	**0**	**-0.1**	0	0	0	-0.4	**-0.1**	**0**	0	0	0	-3.4	**-1.7**	**-1.5**
	③	0	0	0	0	-0.2	0	-8.5	-8.1	-9.6	-0.4	-0.4	-0.3	0	0	0	0	0	0	0	0	0	0	0	0	-10.5	**-8.4**	-9.4
	④	0	0	0	0	-0.1	-0.1	0	0	0	-13.0	**-11.1**	**-11.2**	0	0	**-0.1**	0	0	0	0	0	0	0	0	0	-6.1	-5.5	-5.8
	⑤	0	0	0	-1.1	**-0.3**	**-0.3**	-0.5	**-0.1**	**-0.1**	-1.2	**-0.3**	**-0.5**	-2.4	-2.9	-2.9	0	0	0	0	0	0	0	0	0	-4.1	**-3.0**	-3.3
	⑥	0	0	0	0	-0.1	-0.2	0	0	-0.1	0	-0.2	-0.1	0	0	-0.2	0	-0.2	-0.2	0	-0.1	-0.1	0	0	0	-3.5	-4.1	-4.6
	⑦	0	0	0	-0.1	0	-0.1	0	0	0	-0.1	0	-0.1	0	0	0	0	0	0	-14.9	-14.3	-15.1	0	0	0	-1.2	-1.3	-1.5
	⑧	0	0	0	0	-0.1	0	0	0	0	0	-0.5	**-0.7**	0	0	-0.1	0	0	0	0	0	0	0	-0.1	0	-4.4	-3.9	-5.5

Forcing Pathway

NoCC ***SSP126*** ***SSP370***

*Supplementary Table 4: **Expected relative consumption shock** in % in the affected region (rows) during a p25 yield shock in the shock region (columns) under different SSP and forcing pathways for 2050s. Values in bold represent a significant difference to a NoCC forcing scenario under GCM spread (following a t-test, n=5, alpha = 0.05). Effects are calculated relative to the median (see Methods). ① AMU: Arab Maghreb Union, ② EAC: Eastern African Community, ③ ECCAS: Economic Community of Central African States, ④ ECOWAS: Economic Community of Western African States, ⑤ Egypt, ⑥ RCEAf: Rest of Central Eastern Africa, ⑦ RSouthAf: Rest of Southern Africa, ⑧ SACU: Southern African Customs Union, SEA: Southeast-Asia.*

SSP1 - p25	Shock Region																										
Consumption Region	AMU ①			EAC ②			ECCAS ③			ECOWAS ④			Egypt ⑤			RCEAf ⑥			RSouthAf ⑦			SACU ⑧			SEA		
①	-1.2	-1.0	-1.1	0	0	0	0	0	0	0	0	0	0	0	-0.2	0	0	0	0	0	0	0	0	0	-1.6	**-1.3**	**-1.2**
②	0	0	0	-7.4	-7.5	-6.9	0	0	0	0	0	0	0	0	0	0	0	0	0	0	0	0	0	0	-0.9	**-0.4**	**-0.5**
③	0	0	0	0	0	0	-4.3	-3.4	-3.1	0	0	0	0	0	0	0	0	0	0	0	0	0	0	0	-0.8	-0.9	-0.9
④	0	0	0	0	0	0	0	-0.1	-0.1	-4.3	**-3.4**	**-3.1**	0	0	0	0	0	0	0	0	0	0	0	0	-0.6	-0.5	-0.5
⑤	0	0	0	0	0	0	0	0	0	0	**-0.1**	-0.1	-3.1	-2.4	**-1.7**	0	0	0	0	0	-0.1	0	0	0	-0.3	**-0.7**	**-0.7**
⑥	0	0	0	0	0	0	0	0	0	0	0	0	0	0	0	0	0	0	0	0	0	0	0	0	-0.8	-0.8	-0.8
⑦	0	0	0	0	0	0	0	0	0	0	0	0	0	0	0	0	0	0	-4.4	**-3.6**	**-3.3**	0	0	0	-0.6	-0.5	-0.5
⑧	0	0	0	0	-0.1	0	0	-0.1	-0.2	0	-0.1	-0.2	0	-0.1	0	0	0	0	0	**-0.5**	**-0.5**	0	0	0	-0.4	**-0.9**	**-0.9**

SSP2 - p25	Shock Region																										
Consumption Region	AMU ①			EAC ②			ECCAS ③			ECOWAS ④			Egypt ⑤			RCEAf ⑥			RSouthAf ⑦			SACU ⑧			SEA		
①	-0.4	**-0.8**	-0.5	-0.1	0	0	-0.1	-0.1	0	0	-0.1	-0.1	0	0	0	0	0	0	0	0	0	0	0	0	-1.9	-1.5	-1.9
②	0	0	0	-8.0	-8.2	-7.6	0	0	0	0	0	0	-0.1	-0.1	0	0	0	0	0	0	0	0	0	0	-0.7	**-0.5**	**-0.5**
③	0	0	0	0	0	0	-2.3	-2.2	-2.8	-0.2	**-0.1**	-0.1	0	0	0	0	0	0	0	0	0	0	0	0	-1.1	**-1.0**	-1.3
④	0	0	0	0	0	0	0	0	0	-4.7	**-3.8**	**-3.6**	0	0	0	0	0	0	0	0	0	0	0	0	-0.9	**-0.6**	-1.0
⑤	0	0	0	-0.2	**-0.1**	**0**	0	0	0	-0.1	-0.1	**0**	-1.9	-2.5	-2.2	0	0	0	0	0	0	0	0	0	-0.2	-0.2	-0.2
⑥	0	0	0	0	0	0	0	0	0	0	0	-0.1	0	0	0	-0.6	**-0.2**	**-0.2**	0	0	0	0	0	0	-1.3	-1.0	**-1.1**
⑦	0	0	0	0	0	0	0	0	0	0	0	0	0	0	0	0	0	0	-5.2	**-4.0**	**-3.5**	0	0	0	-0.4	-0.3	-0.3
⑧	0	0	0	0	0	0	0	0	0	-0.3	**-0.1**	**0**	0	0	0	0	0	0	0	0	0	0	0	0	-1.4	**-0.9**	-1.3

SSP3 - p25	Shock Region																										
Consumption Region	AMU ①			EAC ②			ECCAS ③			ECOWAS ④			Egypt ⑤			RCEAf ⑥			RSouthAf ⑦			SACU ⑧			SEA		
①	-0.9	-1.2	-0.9	0	-0.1	0	0	-0.1	0	0	-0.3	-0.2	0	0	0	0	0	0	0	0	0	0	0	0	-1.8	-1.6	-2
②	0	0	0	-6.1	**-7.5**	-6.8	0	0	0	-0.1	0	0	0	0	-0.1	0	0	0	0	0	0	0	0	0	-0.9	**-0.5**	**-0.5**
③	0	0	0	0	0	0	-2.5	-2.3	-2.5	-0.2	-0.1	-0.1	0	0	0	0	0	0	0	0	0	0	0	0	-1.4	-1.5	-1.7
④	0	0	0	0	0	0	0	0	0	-4.5	**-3.8**	**-3.6**	0	0	0	0	0	0	0	0	0	0	0	0	-1	-0.9	-1.1
⑤	0	0	0	-0.1	-0.2	-0.1	0	-0.1	0	-1.2	**-0.2**	**-0.3**	-1.2	-0.8	-1.5	0	0	0	0	0	0	0	0	0	-2.2	**-0.5**	**-0.9**
⑥	0	0	0	0	0	-0.1	0	0	0	0	-0.2	0	0	0	-0.1	0	-0.1	-0.2	0	0	-0.1	0	0	0	-1.1	-1.1	-1.4
⑦	0	0	0	0	0	0	0	0	0	0	0	0	0	0	0	0	0	0	-3.7	-4.1	-4.2	0	0	0	-0.7	**-0.3**	-0.5
⑧	0	0	0	0	0	0	0	0	0	0	-0.2	-0.3	0	0	-0.1	0	0	0	0	0	0	0	0	0	-1.1	-1.2	-1.6

Forcing Pathway

NoCC ***SSP126*** ***SSP370***

Supplementary Table 5: ***Expected relative consumption shock*** *in % in the affected region (rows) during a p75 yield shock in the shock region (columns) under different SSP and forcing pathways for 2050s. Values in bold represent a significant difference to a NoCC forcing scenario under GCM spread (following a t-test, n=5, alpha = 0.05). Effects are calculated relative to the median (see Methods). ① AMU: Arab Maghreb Union, ② EAC: Eastern African Community, ③ ECCAS: Economic Community of Central African States, ④ ECOWAS: Economic Community of Western African States, ⑤ Egypt, ⑥ RCEAf: Rest of Central Eastern Africa, ⑦ RSouthAf: Rest of Southern Africa, ⑧ SACU: Southern African Customs Union, SEA: Southeast-Asia.*

SSP1 - p75	Shock Region																										
	AMU ①			EAC ②			ECCAS ③			ECOWAS ④			Egypt ⑤			RCEAf ⑥			RSouthAf ⑦			SACU ⑧			SEA		
Consumption Region	NoCC	SSP126	SSP370	NoCC	SSP126	SSP370	NoCC	SSP126	SSP370	NoCC	SSP126	SSP370	NoCC	SSP126	SSP370	NoCC	SSP126	SSP370	NoCC	SSP126	SSP370	NoCC	SSP126	SSP370	NoCC	SSP126	SSP370
①	1.0	**0.8**	0.8	0	0	0	0	0	0	0	0.1	0.1	0	0.1	0	0	0	0	0	0	0	0	0	0	0.9	1.0	**1.1**
②	0	0	0	3.6	2.9	2.8	0	0	0	0.1	0	0	0	0	0	0	0	0	0	0	0	0	0	0	0.2	**0.3**	**0.3**
③	0	0	0	0	0	0	1.7	1.5	**1.3**	0.1	0.1	0.1	0	0	0	0	0	0	0	0	0	0	0	0	1.3	**0.9**	1.1
④	0	0	0	0	0	0	0	0	0	4.0	4.2	3.8	0	0	0	0	0	0	0	0	0	0	0	0	0.9	0.8	1.0
⑤	0	0	0	0	0	0	0	0	0	0.1	0.2	0.1	3.41	**2.6**	**2.2**	0	0	0	0	0	0	0	0	0	0.3	0.4	0.5
⑥	0	0	0	0	0	0	0	0	0	0	0	0	0	0	0	0	0.2	0.3	0	0	0	0	0	0	1.0	1.0	1.0
⑦	0	0	0	0.1	**0**	**0**	0	0	0	0.1	**0**	**0**	0	0	0	0	0	0	0.1	0.9	**1.1**	0	0	0	0.3	**0**	**0**
⑧	0	0	0	0	0	0	0	0.1	0	0	**0.4**	0.1	0	0	0	0	0	0	0	0.1	0	0	0	0	0.6	**0.1**	0.5

SSP2 - p75	Shock Region																										
	AMU ①			EAC ②			ECCAS ③			ECOWAS ④			Egypt ⑤			RCEAf ⑥			RSouthAf ⑦			SACU ⑧			SEA		
Consumption Region	NoCC	SSP126	SSP370	NoCC	SSP126	SSP370	NoCC	SSP126	SSP370	NoCC	SSP126	SSP370	NoCC	SSP126	SSP370	NoCC	SSP126	SSP370	NoCC	SSP126	SSP370	NoCC	SSP126	SSP370	NoCC	SSP126	SSP370
①	0.9	0.7	**0.7**	0	0	0	0.1	0	0	0.4	0.2	0.3	0	0	0	0	0	0	0	0	0	0	0	0	1.4	1.1	1
②	0	0	0	5.1	4.1	3.9	0	0	0	0	0	0	0	0	0	0	0	0	0	0	0	0	0	0	0.3	**0.2**	**0.1**
③	0	0	0	0	0	0	2.0	**1.3**	**0.4**	0.1	0.2	0.1	0	0	0	0	0	0	0	0	0	0	0	0	0.6	0.7	0.5
④	0	0	0	0	0	0	0	0	0	4.6	4.4	**3.7**	0	0	0	0	0	0	0	0	0	0	0	0	0.7	0.7	0.7
⑤	0	0	0	0.1	0.1	0.1	0	0	0	0.2	0.1	0.2	1.8	**2.8**	2.4	0	0	0	0	0	0	0	0	0	0.1	0.1	**0.2**
⑥	0	0	0	0	0	0	0	0	0	0	0.1	0.1	0	0	0.1	0	0.2	0.2	0	0	0	0	0	0	0.4	**0.6**	**0.7**
⑦	0	0	0	0	0	0	0	0	0	0	0	0	0	0	0	0	0	0	0.2	**1.1**	**1.1**	0	0	0	0.1	0.1	**0**
⑧	0	0	0	0	0	0	0	0	0	0	0.1	0.1	0	0	0	0	0	0	0	0	0	0	0	0	0.5	0.8	**0.9**

SSP3 - p75	Shock Region																										
	AMU ①			EAC ②			ECCAS ③			ECOWAS ④			Egypt ⑤			RCEAf ⑥			RSouthAf ⑦			SACU ⑧			SEA		
Consumption Region	NoCC	SSP126	SSP370	NoCC	SSP126	SSP370	NoCC	SSP126	SSP370	NoCC	SSP126	SSP370	NoCC	SSP126	SSP370	NoCC	SSP126	SSP370	NoCC	SSP126	SSP370	NoCC	SSP126	SSP370	NoCC	SSP126	SSP370
①	1.2	**0.7**	**1.0**	0	0	0	0.1	0	0	0.5	0.3	**0.1**	0	0	0	0	0	0	0	0	0	0	0	0	1.5	1.2	**1.2**
②	0	0	0	4.5	**3.3**	3.7	0	0	0	0.1	**0**	0.1	0.1	0.2	0.1	0	0	0	0	0	0	0	0	0	0.3	0.4	**0.5**
③	0	0	0	0	0	0	1.5	1.6	1.5	0	0	**0.1**	0	0	0.1	0	0	0	0	0	0	0	0	0	1.0	0.8	1.1
④	0	0	0	0	0	0	0	0	0	4.4	3.9	3.6	0	0	0	0	0	0	0	0	0	0	0	0	0.9	0.7	0.7
⑤	0	0	0	0	0	0	0	0	0	0	0.3	0.1	0	**2.7**	**1.7**	0	0	0	0	0	0	0	0	0	1.2	1.6	1.6
⑥	0	0	0	0	0	0	0	0	0	0	0	0.2	0	0	0.1	0	0	0.4	0	0	0	0	0	0	1.1	0.9	0.7
⑦	0	0	0	0	0.1	0	0	0	0	0	0.2	0	0	0	0	0	0	0	2.0	2.3	2.0	0	0	0	0.8	**0.4**	**0.2**
⑧	0	0	0	0	0.1	0	0	0	0	0	0.3	0	0	0	0	0	0	0	0	0	0	0	0	0	1.1	0.8	0.6

Forcing Pathway

NoCC ***SSP126*** ***SSP370***

*Supplementary Table 6: **Expected relative consumption shock** in % in the affected region (rows) during a p95 yield shock in the shock region (columns) under different SSP and forcing pathways for 2050s. Values in bold represent a significant difference to a NoCC forcing scenario under GCM spread (following a t-test, n=5, alpha = 0.05). Effects are calculated relative to the median (see Methods). ① AMU: Arab Maghreb Union, ② EAC: Eastern African Community, ③ ECCAS: Economic Community of Central African States, ④ ECOWAS: Economic Community of Western African States, ⑤ Egypt, ⑥ RCEAf: Rest of Central Eastern Africa, ⑦ RSouthAf: Rest of Southern Africa, ⑧ SACU: Southern African Customs Union, SEA: Southeast-Asia.*

SSP1 - p95	Shock Region																										
	AMU ①			EAC ②			ECCAS ③			ECOWAS ④			Egypt ⑤			RCEAf ⑥			RSouthAf ⑦			SACU ⑧			SEA		
	NoCC	SSP126	SSP370	NoCC	SSP126	SSP370	NoCC	SSP126	SSP370	NoCC	SSP126	SSP370	NoCC	SSP126	SSP370	NoCC	SSP126	SSP370	NoCC	SSP126	SSP370	NoCC	SSP126	SSP370	NoCC	SSP126	SSP370
Consumption Region ①	2.4	2.0	2.2	0	0	0	0	0	0	0	0.3	0.2	0	0.2	0.2	0	0	0	0	0	0	0	0	0	1.9	1.9	1.8
②	0	0	0	14.8	**5.7**	**6.0**	0	0	0	0.2	0.1	**0.1**	0	0	0	0	0	0	0.1	**0**	**0**	0	0	0	0.8	0.7	**0.6**
③	0	0	0	0.1	**0**	0.1	3.6	**2.7**	2.9	0.4	0.3	0.3	0	0	0.1	0	0	0	0	0	0	0	0	0	1.6	1.5	**1.4**
④	0	0	0	0	0	0	0	0	0	10.7	9.3	8.8	0	0	0	0	0	0	0	0	0	0	0	0	1.7	1.6	1.6
⑤	0	0	0	0.1	**0**	0	0	0	0	0.8	0.7	**0.6**	8.2	**7.0**	**5.5**	0	0	0	0.1	**0**	0.1	0	0	0	0.7	1.0	1.0
⑥	0	0	0	0	0	0	0	0	0	2.2	1.5	1.6	0	0	0.1	1	0.9	0.9	0	0.4	0.4	0	0	0	1.0	1.0	1.2
⑦	0	0	0	0.1	**0**	**0**	0	0	0	0.3	**0**	**0**	0	0	0	0	0	0	5.1	4.4	4.8	0	0	0	0.3	**0**	**0**
⑧	0	0	0	0	0	0	0	0.1	0	0.6	0.4	**0.1**	0	0	0	0	0	0	0.6	**0.1**	0	0	0	0	1.4	**0.9**	1.1

SSP2 - p95	Shock Region																										
	AMU ①			EAC ②			ECCAS ③			ECOWAS ④			Egypt ⑤			RCEAf ⑥			RSouthAf ⑦			SACU ⑧			SEA		
	NoCC	SSP126	SSP370	NoCC	SSP126	SSP370	NoCC	SSP126	SSP370	NoCC	SSP126	SSP370	NoCC	SSP126	SSP370	NoCC	SSP126	SSP370	NoCC	SSP126	SSP370	NoCC	SSP126	SSP370	NoCC	SSP126	SSP370
Consumption Region ①	1.6	1.6	1.4	0.3	**0**	0	0.2	0	**0**	0.6	0.3	0.4	0	0	0	0	0	0	0	0	0	0	0	0	2.2	2.5	1.9
②	0	0	0	16.2	**6.9**	**7.2**	0	0	0	0.1	**0**	**0**	0.1	0.1	0.1	0	0	0	0	0	0	0	0	0	0.6	**0.3**	**0.2**
③	0	0	0	0	0	0	4.0	**2.7**	**2.6**	0.3	0.3	**0.2**	0	**0.1**	0	0	0	0	0	0	0	0	0	0	1.0	1.0	0.8
④	0	0	0	0	0	0	0	0	0	11.0	10.2	9.6	0	0	0	0	0	0	0	0	0	0	0	0	0.9	0.9	1.0
⑤	0	0	0	0.5	**0.2**	**0.2**	0	0	0	0.7	0.7	0.6	4.1	**7.0**	**6.2**	0	0	0	0	0	0	0	0	0	0.5	0.4	0.5
⑥	0	0	0	0	0	0	0	0	0	0	0.2	0.1	0	**0.1**	0.1	0.2	**0.5**	**0.5**	0	0	0	0	0	0	0.8	**1.3**	**1.5**
⑦	0	0	0	0	0	0	0	0	0	0	0	0	0	0	0	0	0	0	5.5	4.7	5.3	0	0	0	0.3	0.1	**0**
⑧	0	0	0	0	0	0	0	0	0	0.5	0.4	0.3	0	0	0	0	0	0	0	0	0	0	0	0	1.9	1.7	1.6

SSP3 - p95	Shock Region																										
	AMU ①			EAC ②			ECCAS ③			ECOWAS ④			Egypt ⑤			RCEAf ⑥			RSouthAf ⑦			SACU ⑧			SEA		
	NoCC	SSP126	SSP370	NoCC	SSP126	SSP370	NoCC	SSP126	SSP370	NoCC	SSP126	SSP370	NoCC	SSP126	SSP370	NoCC	SSP126	SSP370	NoCC	SSP126	SSP370	NoCC	SSP126	SSP370	NoCC	SSP126	SSP370
Consumption Region ①	3.0	**1.8**	**2.0**	0	0	0	0.2	0.1	0	0.9	0.6	**0.3**	0	0	0	0	0	0	0	0	0	0	0	0	2.4	2.2	2.2
②	0	0	0	10.3	**7.9**	**7.7**	0	0	0	0.3	**0.1**	0.2	0.1	0.3	0.2	0	0	0	0	0	0	0	0	0	0.6	0.6	0.6
③	0	0	0	0	0	0	3.3	2.6	2.3	0.1	0	0.2	0	0	**0.1**	0	0	0	0	0	0	0	0	0	1.5	1.6	1.7
④	0	0	0	0	0	0	0	0	0	11.8	9.2	**9.0**	0	**0.1**	0	0	0	0	0	0	0	0	0	0	1.0	1.2	1.2
⑤	0	0	0	0	0	0	0	0	0	0	0.4	0.2	2.4	**7.8**	**5.3**	0	0	0	0	0	0	0	0	0	3.5	**5**	**4.6**
⑥	0	0	0	0	0	0	0	0	0	2.2	1.3	**0.8**	0	0	0.2	0.6	**0.2**	0.6	0	0	0.1	0	0	0	2.2	**1.3**	1.7
⑦	0	0	0	0.1	0.1	**0**	0	0	0	0.7	**0.2**	**0.1**	0	0	0	0	0	0	4.1	3.6	3.7	0	0	0	0.8	0.5	**0.3**
⑧	0	0	0	0	0.1	0	0	0	0	1.1	**0.3**	**0**	0	0.1	0	0	0	0	0	0.1	0	0	0	0	2.1	1.7	**1.4**

Forcing Pathway

NoCC ***SSP126*** ***SSP370***

*Supplementary Table 7: **Expected relative consumption shock** in % in the affected region (rows) during a p100 yield shock in the shock region (columns) under different SSP and forcing pathways for 2050s. Values in bold represent a significant difference to a NoCC forcing scenario under GCM spread (following a t-test, n=5, alpha = 0.05). Effects are calculated relative to the median (see Methods). ① AMU: Arab Maghreb Union, ② EAC: Eastern African Community, ③ ECCAS: Economic Community of Central African States, ④ ECOWAS: Economic Community of Western African States, ⑤ Egypt, ⑥ RCEAf: Rest of Central Eastern Africa, ⑦ RSouthAf: Rest of Southern Africa, ⑧ SACU: Southern African Customs Union, SEA: Southeast-Asia.*

SSP1 - p100 — Shock Region (columns) / Consumption Region (rows)

Consumption Region	AMU ①			EAC ②			ECCAS ③			ECOWAS ④			Egypt ⑤			RCEAf ⑥			RSouthAf ⑦			SACU ⑧			SEA		
①	3.2	2.8	3.1	0	0	0	0	0	0	0.2	0.4	0.5	0	0.2	0.2	0	0	0	0	0	0	0	0	0	2.4	2.7	2.5
②	0	0	0	26.2	**11.6**	**12.6**	0	0	0	0.2	0.2	0.2	0.1	**0**	**0**	0	0	0	0.1	**0**	**0**	0	0	0	1.0	0.8	0.7
③	0	0	0	0.1	**0**	0.1	6.0	**4.9**	5.2	0.6	0.4	0.5	0	0	0.1	0	0	0	0	0	0.1	0	0	0	1.6	1.5	**1.4**
④	0	0	0	0	0	0	0	0	0	15.3	12.5	13.4	0	0	0	0	0	0	0	0	0	0	0	0	1.7	1.7	1.6
⑤	0	0	0	0.1	**0**	0	0	0	0	1.2	**0.8**	1.0	10.5	**8.3**	**7.3**	0	0	0	0.1	**0**	0.1	0	0	0	0.7	**1.3**	**1.4**
⑥	0	0	0	0	0	0.1	0	0	0	5.1	**2.8**	3.8	0	0.1	0.1	1.0	1.0	1.0	0.3	**0.8**	0.7	0	0	0	1.1	**1.8**	1.6
⑦	0	0	0	0.1	**0**	**0**	0	0	0	0.3	**0**	**0**	0	0	0	0	0	0	9.2	**7.4**	7.2	0	0	0	0.3	**0**	**0**
⑧	0	0	0	0	0	0	0	0.1	0	0.6	0.4	**0.1**	0	0	0	0	0	0	0.6	**0.1**	0	0	0	0	1.4	1.2	1.2

SSP2 - p100 — Shock Region (columns) / Consumption Region (rows)

Consumption Region	AMU ①			EAC ②			ECCAS ③			ECOWAS ④			Egypt ⑤			RCEAf ⑥			RSouthAf ⑦			SACU ⑧			SEA		
①	2.4	2.0	1.7	0.3	**0**	0	0.3	0	**0**	0.9	0.5	0.7	0	0	0	0	0	0	0	0	0	0	0	0	2.9	2.7	2.6
②	0	0	0	27.5	**12.8**	**13.5**	0	0	0	0.2	**0.1**	**0.1**	0.1	0.1	0.1	0	0	0	0	0	0	0	0	0	0.6	**0.3**	**0.3**
③	0	0	0	0	0	0	6.4	**4.9**	5.0	0.4	0.4	0.3	0	**0.1**	0.1	0	0	0	0	0	0	0	0	0	1.3	1.4	1.1
④	0	0	0	0	0	0	0	0	0	16.1	13.6	14.7	0	0	0	0	0	0	0	0	0	0	0	0	1.1	1.1	1.2
⑤	0	0	0	0.5	**0.2**	**0.2**	0.1	**0**	**0**	0.8	0.8	0.9	6.0	**8.1**	7.9	0	0	0	0	0	0	0	0	0	0.5	0.6	0.6
⑥	0	0	0	0	0	0.1	0	0	0	0	0.9	1.2	0	0.1	0.1	0.8	0.7	0.7	0	0	0	0	0	0	1.8	1.6	1.7
⑦	0	0	0	0	0	0	0	0	0	0.1	0.1	**0**	0	0	0	0	0	0	9.3	7.8	**7.9**	0	0	0	0.3	0.1	**0**
⑧	0	0	0	0	0	0	0	0	0	0.5	0.5	0.5	0	0	0	0	0	0	0	0	0	0	0	0	1.9	2.0	2.1

SSP3 - p100 — Shock Region (columns) / Consumption Region (rows)

Consumption Region	AMU ①			EAC ②			ECCAS ③			ECOWAS ④			Egypt ⑤			RCEAf ⑥			RSouthAf ⑦			SACU ⑧			SEA		
①	3.7	**2.3**	**2.5**	0	0	0	0.3	**0.1**	**0**	0.9	0.7	0.7	0	0	0	0	0	0	0	0	0	0	0	0	2.9	2.7	2.6
②	0	0	0	18.3	**12.0**	**12.8**	0	0	0	0.3	**0.1**	0.2	0.2	0.3	0.3	0	0	0	0	0	0	0	0	0	0.7	0.7	0.7
③	0	0	0	0	0	0	5.2	4.6	4.7	0.3	0.1	0.3	0	0	**0.1**	0	0	0	0	0	0	0	0	0	1.5	1.6	1.8
④	0	0	0	0	0	0	0	0	0	16.9	12.3	13.2	0	**0.1**	0.1	0	0	0	0	0	0	0	0	0	1.4	1.3	1.4
⑤	0	0	0	0	0	0	0	0	0	0	0.6	0.5	4.1	**9.5**	**7.3**	0	0	0	0	0	0	0	0	0	5.7	5.9	5.5
⑥	0	0	0	0	0	0	0	0	0	4.4	2.4	2.9	0	0	0.2	1.1	**0.5**	0.7	0	0	0.1	0	0	0	3.3	**2.2**	**1.7**
⑦	0	0	0	0.3	0.1	**0**	0	0	0	0.7	**0.3**	**0.2**	0	0	0	0	0	0	8.7	**6.1**	**6.0**	0	0	0	0.8	0.6	**0.4**
⑧	0	0	0	0	0.1	0	0	0	0	1.1	**0.3**	**0.2**	0	0.1	0	0	0	0	0	0.1	0	0	0	0	2.2	2.0	1.8

Forcing Pathway

NoCC *SSP126* *SSP370*

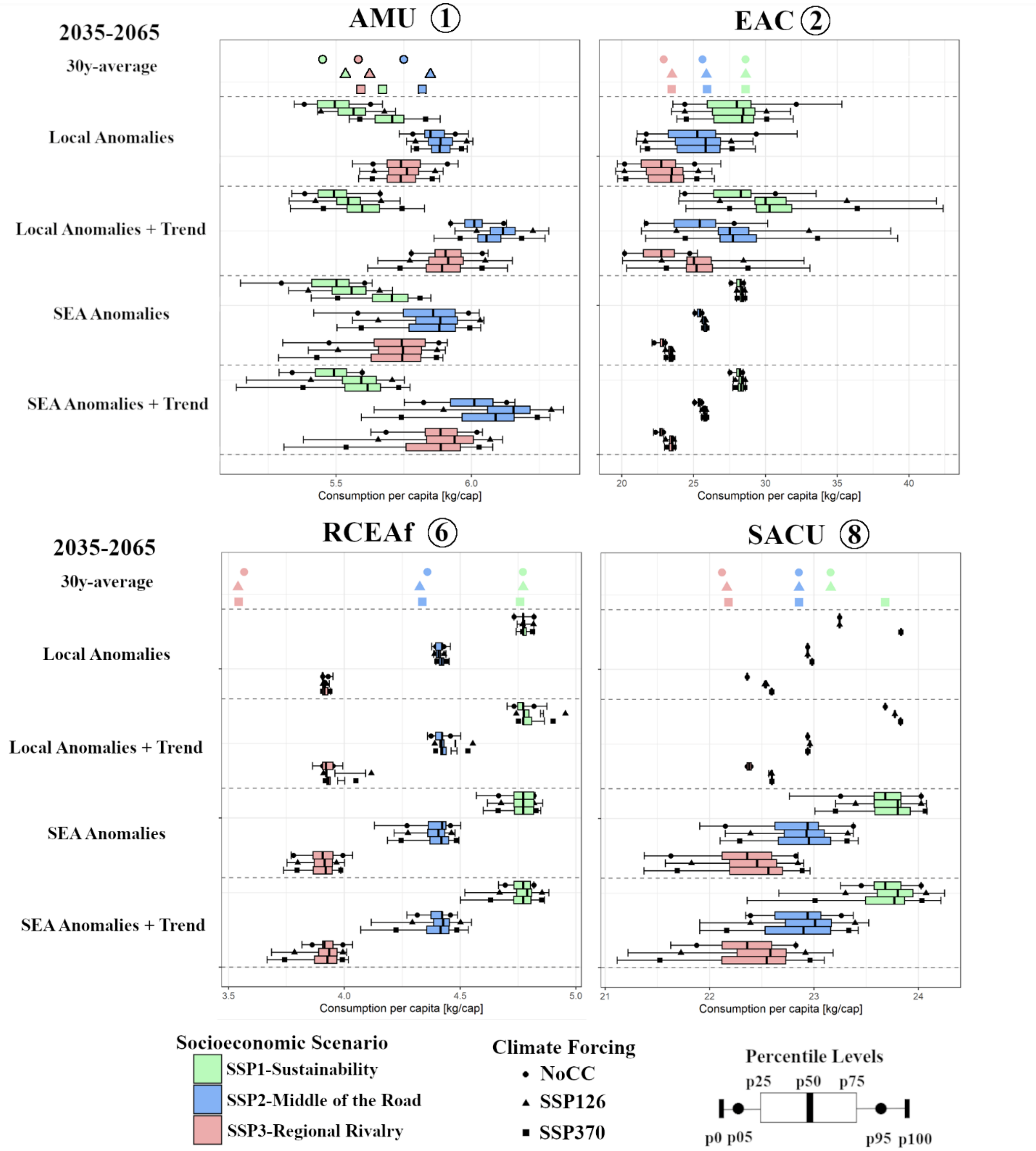

Supplementary Figure 8: ***Predicted ensemble rice consumption per capita percentile levels*** *in kg/pers for the AMU, EAC, RCEAf, and SACU regions under different socioeconomic and climatic narratives for the 2035-2065 time window (n=30). 30y-average values indicate effects on rice availability (see Gradual Changes). ① AMU: Arab Maghreb Union, ② EAC: Eastern African Community, ⑥ RCEAf: Rest of Central Eastern Africa, ⑧ SACU: Southern African Customs Union. See Methodology for the calculation of the distribution.*

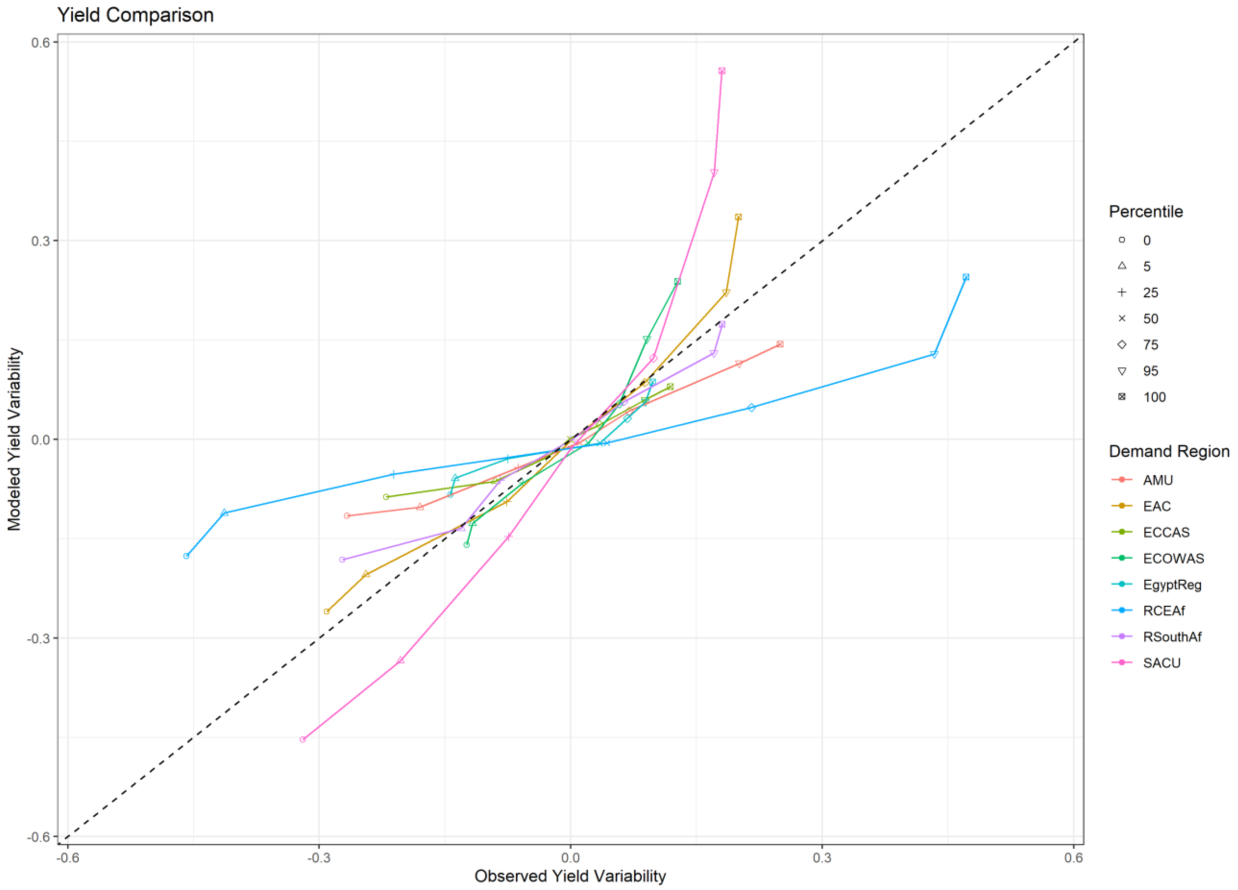

Supplementary Figure 9: ***Comparison Q-Q plot*** *of the observed relative Yield Variation (coming from FAOSTAT) and the modeled yield variation (coming from EPIC-IIASA) for the different African regions in the 1985-2015 time window. This comparison indicates that the modeled yield variation underestimates the effects of the lower extremes.*
