# Supplementary material for: Rice availability and stability in Africa under future socio-economic development and climatic change
Source: Nat Food. 2023 Jun 19;4(6):518–27. doi: 10.1038/s43016-023-00770-5 (PMC10289898; doi:10.1038/s43016-023-00770-5)
Supplement: Supplementary file 2 — Reporting Summary [file 43016_2023_770_MOESM2_ESM.pdf]

nature portfolio

Corresponding author(s): Koen De Vos

Last updated by author(s): May 2, 2023

Reporting Summary

Nature Portfolio wishes to improve the reproducibility of the work that we publish. This form provides structure for consistency and transparency in reporting. For further information on Nature Portfolio policies, see our Editorial Policies and the Editorial Policy Checklist.

Statistics

For all statistical analyses, confirm that the following items are present in the figure legend, table legend, main text, or Methods section.

n/a	Confirmed	
☐	☒	The exact sample size (*n*) for each experimental group/condition, given as a discrete number and unit of measurement
☒	☐	A statement on whether measurements were taken from distinct samples or whether the same sample was measured repeatedly
☐	☒	The statistical test(s) used AND whether they are one- or two-sided<br>*Only common tests should be described solely by name; describe more complex techniques in the Methods section.*
☒	☐	A description of all covariates tested
☒	☐	A description of any assumptions or corrections, such as tests of normality and adjustment for multiple comparisons
☐	☒	A full description of the statistical parameters including central tendency (e.g. means) or other basic estimates (e.g. regression coefficient) AND variation (e.g. standard deviation) or associated estimates of uncertainty (e.g. confidence intervals)
☐	☒	For null hypothesis testing, the test statistic (e.g. *F*, *t*, *r*) with confidence intervals, effect sizes, degrees of freedom and *P* value noted<br>*Give P values as exact values whenever suitable.*
☒	☐	For Bayesian analysis, information on the choice of priors and Markov chain Monte Carlo settings
☒	☐	For hierarchical and complex designs, identification of the appropriate level for tests and full reporting of outcomes
☒	☐	Estimates of effect sizes (e.g. Cohen's *d*, Pearson's *r*), indicating how they were calculated

Our web collection on statistics for biologists contains articles on many of the points above.

Software and code

Policy information about availability of computer code

Data collection	GAMS version 32.2
Data analysis	R version 4.0.0

For manuscripts utilizing custom algorithms or software that are central to the research but not yet described in published literature, software must be made available to editors and reviewers. We strongly encourage code deposition in a community repository (e.g. GitHub). See the Nature Portfolio guidelines for submitting code & software for further information.

Data

Policy information about availability of data

All manuscripts must include a data availability statement. This statement should provide the following information, where applicable:

- Accession codes, unique identifiers, or web links for publicly available datasets
- A description of any restrictions on data availability
- For clinical datasets or third party data, please ensure that the statement adheres to our policy

The datasets generated during and/or analysed during the current study are available from the corresponding author on request.

Field-specific reporting

Please select the one below that is the best fit for your research. If you are not sure, read the appropriate sections before making your selection.

☐ Life sciences ☒ Behavioural & social sciences ☐ Ecological, evolutionary & environmental sciences

For a reference copy of the document with all sections, see nature.com/documents/nr-reporting-summary-flat.pdf

Behavioural & social sciences study design

All studies must disclose on these points even when the disclosure is negative.

Study description	This is a modelling-based study that analyses quantitative simulation data generated with a spatially explicit, economic partial equilibrium model of the agriculture, forestry and bioenergy sectors. The study focuses in specific on agricultural production, consumption, and trade at African country and region level.
Research sample	The study sample consists of all 54 African countries, which are grouped into 8 regions similar to Janssens et al. 2022). The study uses existing open source dataset to calibrate the partial equilibrium land use model to observed trends in 2000 - 2020 and then simulates potential future pathways up to 2050. Datasets used as model input are from the EPIC-IIASA crop model (Folberth et al. 2020) and from the online SSP-repository.<br><br>Folberth, C. et al. The global cropland-sparing potential of high-yield farming. Nat. Sustain. 3, 281–289 (2020).<br>Janssens, C. et al. A sustainable future for Africa through continental free trade and agricultural development. Nature Food 3, 608-618 (2022)
Sampling strategy	Sample size corresponds to full population (African countries)
Data collection	The data was generated with a partial equilibrium model and the GAMS software.
Timing	The model simulations were generated between 25-01-2022 and 04-03-2023 including model reruns during the reviewing process
Data exclusions	No data were excluded from the analysis.
Non-participation	No participants dropped out of the study.
Randomization	Not applicable.

Reporting for specific materials, systems and methods

We require information from authors about some types of materials, experimental systems and methods used in many studies. Here, indicate whether each material, system or method listed is relevant to your study. If you are not sure if a list item applies to your research, read the appropriate section before selecting a response.

Materials & experimental systems

n/a	Involved in the study	
☒	☐	Antibodies
☒	☐	Eukaryotic cell lines
☒	☐	Palaeontology and archaeology
☒	☐	Animals and other organisms
☒	☐	Human research participants
☒	☐	Clinical data
☒	☐	Dual use research of concern

Methods

n/a	Involved in the study	
☒	☐	ChIP-seq
☒	☐	Flow cytometry
☒	☐	MRI-based neuroimaging
